# Pilot study to evaluate the use of remote patient monitoring to guide the timing of valve intervention in patients with severe asymptomatic aortic stenosis (APRAISE-AS): study protocol for a randomised controlled trial delivered in two tertiary cardiac centres in the UK

**DOI:** 10.1136/bmjopen-2024-086587

**Published:** 2024-06-10

**Authors:** Nazish Khan, Richard P Steeds, Derek Kyte, Larissa Fabritz, Philip Collis, Winnie Chua, Clive Stubbs, Samir Mehta, Yongzhong Sun, Mary Nulty, Katie Kirkham, James M Cotton

**Affiliations:** 1 Department of Cardiology (QEHB), University Hospitals Birmingham NHS Foundation Trust, Birmingham, UK; 2 Institute of Cardiovascular Sciences, University of Birmingham, Birmingham, UK; 3 School of Allied Health and Community, University of Worcester, Worcester, UK; 4 Centre for Patient Reported Outcomes Research, Institute for Applied Health Research, University of Birmingham, Birmingham, UK; 5 University Center of Cardiovascular Sciences & Department of Cardiology, University Heart and Vascular Center, University Hospital Hamburg Eppendorf, Hamburg, Germany; 6 Birmingham Clinical Trials Unit, Institute for Applied Health Research, University of Birmingham, Birmingham, UK; 7 Department of Cardiology, The Royal Wolverhampton Hospitals NHS Trust, Wolverhampton, UK

**Keywords:** Patient Reported Outcome Measures, Valvular heart disease, Patient-Centered Care, Patient Satisfaction

## Abstract

**Introduction:**

Aortic stenosis (AS) is common affecting >13% of adults over the age of 75 years. In people who develop symptoms, without valve replacement, prognosis is dismal with mortality as high as 50% at 1 year. In asymptomatic patients, the timing of valve intervention is less well defined and a strategy of watchful waiting is recommended. Many, however, may develop symptoms and attribute this to age related decline, rather than worsening AS. Timely intervention in asymptomatic severe AS is critical, since delayed intervention often results in poor outcomes. Proactive surveillance of symptoms, quality of life and functional capacity should enable timely identification of people who will benefit from aortic valve replacement. There are no data however, to support the clinical and cost effectiveness of such an approach in a healthcare setting in the UK. The aim of this pilot trial is to test the feasibility of a full-scale randomised controlled trial (RCT) to determine the utility of proactive surveillance in people with asymptomatic severe AS to guide the timing of intervention.

**Methods and analysis:**

APRAISE-AS is a multi-centre, non-blinded, two-arm, parallel group randomised controlled trial of up to 66 participants aged >18 years with asymptomatic severe AS. Participants will be randomised to either standard care or standard care supplemented with the APRAISE-AS intervention. Primary outcomes will capture; adherence to and participant acceptability of the intervention, recruitment and retention rates, and completeness of data collection. The findings will be used to inform the sample size and most appropriate outcome measure(s) for a full-scale RCT and health economic evaluation.

**Ethics and dissemination:**

Ethical approval was granted by the Black Country REC, reference: 22/WM/0214. Results will be submitted for publication in peer-reviewed journals and disseminated at local, regional and national meetings where appropriate.

**Trial registration number:**

ISRCTN19413194 registered on 14.07.2023.

**Strengths and limitations of this study**

- The study uses a randomised controlled trial (RCT) design to assess the feasibility of how subjective (symptoms and health-related quality of life) and objective (functional capacity) patient reported data might supplement standard care to inform the timeliness of aortic valve replacement.
- The intervention has been developed via a process of participatory design in collaboration with patients and clinicians.
- The intervention provides an innovative way of using digital technology in patients with heart valve disease (aortic stenosis), to support patient engagement and improve the patient pathway through facilitating enhanced remote patient monitoring, near real time clinician assessment and risk stratification to inform the optimum time at which valve intervention should take place.
- The study is not powered to determine clinical outcomes but is designed to determine the feasibility of conducting a full-scale RCT to understand the clinical utility and cost-effectiveness of integrating an innovative approach to proactive, remote patient monitoring into the clinical management pathway of people with asymptomatic severe aortic stenosis.

## Introduction

Heart valve disease (HVD) affects 1.5 million people over the age of 65 years in the UK and is an important cause of morbidity and mortality.^1^ Aortic stenosis (AS) is the most common acquired form of HVD encountered in clinical practice.^2^ The overall burden of disease in the general population is substantial with an incidence as high as 13% in those aged ≥75 years.^3^ Prevalence and associated healthcare costs secondary to AS are likely to rise in view of an increasingly ageing, frail and multi-morbid population.

AS is characterised by insidious thickening and calcification of a native aortic valve and is an active process driven by lipid accumulation, inflammation and fibrosis.^4 5^ While the underlying pathophysiology of the condition is similar to the process of atherosclerosis, there are no proven medical treatments that can limit disease progression and AS is treated by aortic valve replacement (AVR).^2^

Severity of AS is determined by echocardiographic parameters including the maximal velocity, mean pressure gradient and aortic valve area.^6^ Intervention is mandated in severe AS following the development of symptoms including breathlessness, dizziness, blackouts, chest pain and palpitations. Prognosis is dismal following the development of symptoms, with 1-year mortality as high as 50%, and prompt intervention is therefore critical.^6 7^

In asymptomatic patients however, the timing of valve intervention remains less well defined. A strategy of watchful waiting is adopted, and patients are counselled on the importance of immediate reporting should symptoms develop. Regular clinical surveillance is mandatory and a full physical assessment and echocardiogram should be undertaken at least every 6 months.^6^ The slowly progressive nature of AS combined with the advanced age of the affected population, however, predispose to under-reporting and/or underestimation of symptoms.^8^ Risks associated with a watchful waiting approach include irreversible myocardial damage, sudden cardiac death (with an incidence of 1.5% per year), delayed symptom reporting and dying while awaiting valvular intervention.^9 10^

### Unmet need

The optimal timing of valve intervention in asymptomatic severe AS is currently under investigation in a number of randomised controlled clinical trials; the outcomes of EARLY-TAVR and EVOLVED, will provide insight into whether proactive surveillance, diagnostic imaging and an understanding of the optimum window of opportunity can be implemented in clinical practice to guide the timing of intervention in patients with asymptomatic severe AS.^11 12^

### Objective

To date there have been no high-quality, multicentre randomised controlled trials (RCTs) to test the clinical and cost-effectiveness of whether proactive symptom surveillance and remote patient monitoring using a software application (‘app’) in addition to a traditional ‘watchful waiting’ strategy in adult patients with asymptomatic severe AS allows for earlier identification of symptoms and timelier aortic valve intervention leading to improved clinical outcomes. However, before a large RCT can be undertaken, a pilot trial is required to inform the most appropriate outcome measures and sample size for a full-scale RCT. Therefore the APRAISE-AS study will pilot the trial protocol, in order to assess the feasibility and potential refinement of the trial design and/or trial intervention prior to undertaking a larger RCT investigating whether proactive reporting of symptoms, definitive disease specific patient-reported outcome measures (PROMs) and assessment of frailty in addition to standard care can be used to help inform the timeliness of valve intervention in patients with asymptomatic severe AS.

## Methods

### Trial design and setting

**Figure 1:**
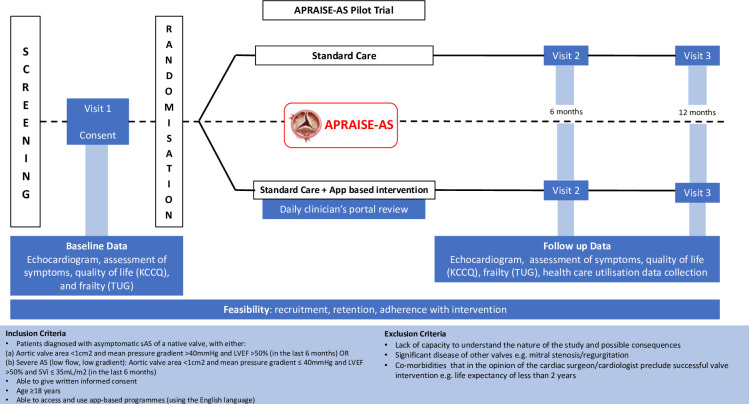
APRAISE-AS trial schema/flow chart. AS, aortic stenosis; KCCQ, Kansas City Cardiomyopathy Questionnaire; LVEF, left ventricular ejection fraction; TUG, timed up and go.

This protocol adheres to guidance from the Standard Protocol Items: Recommendations for Interventional Trials (SPIRIT) and a SPIRIT checklist is included in an accompanying online supplemental file.^13 14^ The APRAISE-AS pilot trial is a multi-centre, non-blinded, two-arm, parallel group randomised controlled pilot trial of up to 66 participants aged 18 years or over with asymptomatic severe AS. The pilot trial will be conducted in two tertiary cardiology and cardiothoracic surgery heart valve centres in the West Midlands. Participants will be randomised in a 1:1 ratio to receive either standard care or standard care supplemented with the APRAISE-AS intervention. The study trial schema is described in figure 1.

10.1136/bmjopen-2024-086587.supp1 Supplementary data

### Recruitment and eligibility

Recruitment commenced in February 2023 in The Royal Wolverhampton NHS Trust and October 2023 at the Queen Elizabeth Hospital Birmingham and closed in January 2024 and February 2024, respectively. Patients were invited to participate if they had a diagnosis of asymptomatic severe AS and if they met the following eligibility criteria.

### Inclusion criteria

- Patients diagnosed with asymptomatic severe AS of a native valve, in line with current international guideline and defined as either:
  - Aortic valve area <1 cm^2^ and mean pressure gradient >40 mm Hg and left ventricular ejection fraction (LVEF) >50% (in the last 6 months) or
  - Aortic valve area <1 cm^2^ and mean pressure gradient ≤40 mm Hg, stroke volume index ≤35 mL/m^2^ and LVEF >50% (in the last 6 months).
- Able to give written informed consent.
- Age ≥18 years.
- Able to access and use app-based programmes.

### Exclusion criteria

- Lack of capacity to understand the nature of the study and possible consequences as determined by their attending clinician.
- Moderate or more severe disease of other valves for example, mitral stenosis/regurgitation.
- Comorbidities that in the opinion of the cardiac surgeon/cardiologist preclude successful valve intervention for example, life expectancy of less than 2 years.

### Participation identification, recruitment and informed consent

Adult patients with a confirmed diagnosis of severe asymptomatic AS of a native valve were recruited. Patients were identified for eligibility and approached for recruitment via one of three routes: (i) following automated email alert for people who meet echocardiographic parameters associated with severe AS, (ii) new referrals seen in outpatient clinics or following identification at ward level, or (iii) from cardiac investigation department echocardiogram diagnostic lists. Existing patients known to the respective cardiac research teams were also be approached for recruitment. Following confirmation of eligibility by the cardiovascular research team (clinicians/pharmacists/nurses), potential participants were contacted by telephone to gauge interest in the trial and request permission for a participant information sheet to be sent to them either via email or by post. In addition, potential participants were also be given a participant information sheet during their clinic appointment. The research team gave potential participants sufficient time to review the information provided, discuss the trial with family members and/or their general practitioner. The chief investigator/site principal investigators ensured that participants fully understand the aim of the trial, the intervention and potential benefits and drawbacks associated with participation. On receiving an expression of interest from the potential participant, they were invited to an outpatient appointment where informed consent for participation was obtained in addition to completion of baseline assessments of symptom status, functional capacity and quality of life were undertaken prior to randomisation. Participants were reminded that participation is voluntary and they may withdraw from the trial at any time and it will not affect the care they receive.

### Randomisation

Following baseline assessments, participants were randomised at the level of the individual in a 1:1 ratio to either standard care (control arm) or standard care supplemented with the APRAISE-AS intervention (intervention arm). Randomisation was undertaken by Birmingham Clinical Trials Unit (BCTU) using a computer-generated sequence created by a programmer independent to the study team. A minimisation algorithm has been used within the randomisation system to ensure balance in the intervention allocations over the following variables:

- Age (‘<50 years’ and ‘≥ 50 years’).
- Sex (‘male’ and ‘female’).
- Ethnicity (‘white’ and ‘non-white’).

To avoid the possibility of the intervention allocation becoming predictable, a random element will be included in the algorithm (unspecified here), so that each participant has a chance of being randomised to the opposite intervention that they would have otherwise received.

### Interventions

Participants assigned to standard care (control arm) will continue to receive usual care which includes a 6 monthly valve clinical review and surveillance echocardiogram (as part of a watchful waiting strategy). For participants assigned to the intervention arm, standard care will be supplemented with the APRAISE-AS intervention. The APRAISE-AS intervention includes an app and a clinician’s portal which has been developed via a process of participatory design in collaboration with our patients and clinicians, which will collect both subjective and objective data on a patients’ health and well-being following their diagnosis of asymptomatic severe AS. This information will be presented to the healthcare team via a bespoke platform which allows near real-time surveillance of the patients’ healthcare data (Clinician’s Portal). The aim of the app is to facilitate clinical prioritisation of patients who demonstrate symptoms or early signs of decompensation to allow for escalation of treatment and/or earlier intervention. These self-reported patient tests will be used to supplement routine clinical appointments.

For participants randomised to standard care plus the APRAISE-AS intervention, a member of the research team will install and provide access to the app on the participant’s own, or a Trust sourced, device. Over a 12-month period, participants will use the app to self-report information relating to:

- Symptoms ((assessed using New York Heart Association and Canadian Cardiovascular System classification) dyspnoea/fatigue/palpitations/angina and syncope) on a weekly basis.
- Frailty/functional capacity (timed up and go test) every 2 weeks (frailty, assessed using gait speed, is known to correlate with major morbidity and mortality after valve surgery and provides a continuous reproducible measure allowing for an assessment of functional status over time^15–17^).
- Disease specific PROMs using the Kansas City Cardiomyopathy Questionnaire (KCCQ) once a month (KCCQ has been studied extensively in the setting of AS and has been found to be a reliable, reproducible and valid measure of symptoms, functional status, quality of life and prognosis^18^).

Participants will receive automated reminders prior to submitting a self-assessment via the app and 24 hours after a failure to submit their report, if necessary. Severe symptoms noted by the patient will trigger an email alert to the APRAISE-AS clinical team (dedicated monitored email inbox) and a simultaneous patient notification advising them to make contact with their clinical team during office hours (or to use standard NHS support mechanisms outside of these hours). The clinical team will monitor for notifications and respond in line with standard care recommendations during office hours. Actions taken in response to an alert will be logged in the patient’s electronic healthcare record by a member of the APRAISE-AS clinical team (including members of the cardiovascular research team).

### Outcome measures and study procedures

The primary aim of this study is to pilot the trial protocol and assess the feasibility and potential refinement of the trial design prior to undertaking a larger RCT investigating whether proactive surveillance/reporting of symptoms, definitive disease specific PROMs and assessment of frailty in addition to standard care can be used to help inform the timeliness of valve intervention in patients with asymptomatic severe AS. In addition, the pilot will allow for refinement of the trial design and/or trial intervention prior to undertaking a larger multi-centre RCT which will assess the clinical and cost-effectiveness of remote patient monitoring in this setting. The aim of using the outcomes to enable pathway transformation and service redesign through the incorporation of remote monitoring into the clinical management pathway for people with asymptomatic severe AS.

The pilot trial will:

- Test and pilot the trial protocol (including recruitment and retention rates, data collection processes, data completeness and adherence to the APRAISE-AS intervention).
- Inform selection of the most appropriate primary outcome measure for a full-scale multicentre RCT.
- Provide data to help estimate the sample size for a full-scale multicentre RCT.
- Provide a platform to develop and pilot the processes to capture costs and outcomes to inform the health economic evaluation for a full-scale multicentre RCT.
- Determine key participation criteria for centre involvement in a full-scale RCT.

### Primary outcomes

In addition to piloting the trial protocol, the pilot study will assess:

- The utility of the ‘app’ using quantitative (eg, usage data, data completeness) and qualitative (interviews) data.
- Engagement with and acceptability of the intervention.
- Recruitment rate (per month).
- Number of withdrawals and losses to follow-up (with reasons).

### Secondary outcomes

Data will be collated on outcomes measures that we plan to collect in the definitive RCT (eg, primary outcome in the phase III trial at 12-month follow-up). While the pilot trial is not powered to detect meaningful differences in these outcomes, collection of these data will provide insight into how readily they can be collected in the context of a larger multicentre RCT. Data will be collected on:

- Disease-specific PROMs assessed using KCCQ.
- Frailty/functional capacity measured using the timed up and go test.
- Time to heart valve replacement.
- In-hospital mortality in those who undergo valve replacement.
- Mortality at 30 days postrandomisation.
- Mortality at 1 year postrandomisation.
- Incidence of stroke in those who undergo valve replacement.
- Incidence of myocardial infarction n those who undergo valve replacement.
- Left ventricular ejection fraction at 6 and 12 months.
- Incidence of unplanned hospital admissions.

All study staff and participants will be invited to complete a trial process questionnaire at the end of the study, which will evaluate aspects surrounding:

- The extent and frequency of data collection.
- Randomisation procedure.
- Acceptability of the intervention (eg, for patients using the mobile app rating score tool).
- Appropriateness of the frequency of APRAISE-AS reporting, alert thresholds and subsequent management.
- The utility of the clinician’s portal and embedding remote monitoring into the clinical management pathway.

### Study procedures

During the baseline visit participants’ demographic (age, gender and ethnicity) and biomarker (creatinine, creatinine clearance) data will be collected. Baseline assessment of symptoms, functional capacity (using the timed up and go test) and health-related quality of life (using the disease specific KCCQ) will be undertaken. Undertaking an objective assessment of frailty using a parameter such as gait speed, which correlates with major morbidity and mortality after valve surgery, provides a continuous reproducible measure allowing for an assessment of functional status over time.^15–17^ In addition, a comprehensive determination of health-related quality of life for patients undergoing valvular intervention, incorporating heart failure specific measures is recommended.^15^ The disease specific PROM KCCQ has been studied extensively in the setting of AS and has been found to be a reliable, reproducible and valid measure of symptoms, functional status, quality of life and prognosis.^18^

As described in table 1, all participants will complete the timed up and go test and paper versions of the KCCQ and symptom tracker questionnaires (in clinic) at baseline (prior to randomisation), and when attending the two subsequent follow-up visits. For participants assigned to the standard care arm, postrandomisation timed up and go, symptom and health status questionnaires will only be completed at the routine 6-month and 12-month follow-up appointments. For those participants assigned to the intervention arm, this information will also be collected via the app at prespecified frequencies postrandomisation as well as during their routine 6-month and 12month follow-up appointments.

**Table 1:** Schedule of assessments for Kansas City Cardiomyopathy Questionnaire (KCCQ) (patient-reported outcome measures), symptoms and timed up and go (frailty) data capture

Assessment	Baseline/prior to randomisation	Once a week±2 days	Once every 2 weeks±2 days	Once a month±2 days	Month 6±2 days	Month 12±2 days	Ad-hoc reporting	Clinical site continuous daily monitoring (intervention arm)	Responding to severe symptoms* clinical site continuous daily monitoring
Control group									
Symptoms	x				x	x			x
Timed up and go	x				x	x			x
KCCQ	x				x	x			x
Intervention group									
Symptoms	x	x			x	x	x	x	x
Timed up and go	x		x		x	x	x	x	x
KCCQ	x			x	x	x	x	x	x

*Following submission of a severe symptom, the clinical team will document the action taken and outcome within the notes section in the clinician’s portal and within the patient record used as per standard local clinical practice.

Adherence with the APRAISE-AS intervention will be assessed by calculating the number and percentage (with 95% CI) of patients who complete the symptom surveillance, frailty and quality of life assessments as scheduled. To be considered adherent, patients will need to have submitted their report within 24 hours of the scheduled time-point. Incomplete submissions (ie, with some questions not answered) will be accepted for the purpose of measuring adherence. Ad-hoc reports (ie, those completed outside the scheduled reporting periods) will not contribute to the assessment of adherence, although we will assess the number of APRAISE-AS reports completed by each patient.

### Sample size and justification

Since this is a pilot study a formal sample size calculation has not been performed. In order to obtain estimates of the parameters needed for sample size determination, in line with published recommendations for pilot studies, 30 patients are required.^19 20^ Assuming a loss to follow-up rate of 10% over the 12-month follow-up period, the study will require 33 patients to be randomised to each group, with a total of 66 patients overall. This will also allow for estimation of recruitment and retention rates with 95% CI maximum widths of 20% and 25%, respectively.

### Analysis of outcome measures

A separate Statistical Analysis Plan will be produced and will provide a more comprehensive description of the planned statistical analyses.

The primary comparison groups will be composed of those randomised to standard care supplemented with the APRAISE-AS intervention (intervention group) versus those randomised to standard care (control group). All analyses will be based on the intention to treat principle, that is, all patients will be analysed in the treatment group to which they were randomised irrespective of compliance with the allocated treatment or other protocol deviation. The data analysis for this pilot trial will mainly be descriptive and focus on CI estimation, with no hypothesis testing performed. The reporting and presentation of this trial will be in accordance with the Consolidated Standards of Reporting Trials guidelines for randomised trials.

The pilot data will also help inform the selection of the most appropriate primary outcome measure for the main RCT and provide contributory data to facilitate estimation of the sample size required for the main RCT. Analysis methods will be chosen according to the data type of the outcome under investigation, in brief:

- *Continuous outcomes (eg, quality of life*): these data will be summarised using means and SD, with differences in means with 95% CIs reported. Longitudinal plots of the data over time will also be constructed for visual presentation of the data.
- *Categorical (dichotomous) outcomes (eg, hospitalisation rates*): the number of patients and percentages experiencing the event will be summarised between groups.
- *Time to event outcomes (eg, time to symptom development, valve replacement, mortality):* the numbers of patients and percentages experiencing the event will be summarised over time between groups. Kaplan-Meier survival curves will be constructed for visual presentation of time-to-event data.

### Missing data and sensitivity analyses

Every attempt will be made to collect full follow-up data on all study patients; it is thus anticipated that missing data will be minimal. The assessment of missing data is an outcome measure of this pilot trial and forms part of the assessment of the feasibility of a future trial. The amount of missing data at outcome measure level will also be considered when assessing and identifying a suitable primary outcome for the future trial. As this is a pilot trial, no formal sensitivity analysis will be conducted.

### Planned interim analysis

Since this is a pilot study a planned interim analysis will not be undertaken. However, the trial oversight committee will access to recruitment, retention and data collection information.

### Planned final analysis

The primary analysis for the study will occur once all patients have completed their 12-month assessment and corresponding outcome data have been entered onto the study database (REDCap) and validated as being ready for analysis. This analysis will include data items up to and including the 12-month assessment and no further.

### Health economics

A health economic evaluation is not planned for this pilot trial. However, data will be collected to capture costs and outcomes to inform the health economic evaluation for a multi-centre full-scale RCT. Data will be collated in relation to cardiology clinical staff time and activity in response to APRAISE-AS notifications, including ad-hoc telephone consultations, inpatient’s hospitalisations and onward referrals for consideration of AVR, in addition to NHS costs attributable to maintenance of the APRAISE-AS clinician’s portal. Where possible data on healthcare resource utilisation will be collected from both electronic patient records and case report forms (CRF) either during study follow-up visits or following response to a severe symptom alert.

### Monitoring, trial management and oversight

The chief investigator has overall responsibility for the study. The Trial Management Group (TMG) consisting of the cardiovascular research team (including chief investigator and site principal investigators) will oversee day to day running of trial. Due to the small sample size and pilot nature of this trial, the Trial Steering and Data Monitoring Committees have been combined into a single Trial Oversight Committee (TOC). The TOC will provide overall oversight of the trial, including the practical aspects of the study, as well as ensuring that the study is run in a way which is both safe for the patients and provides appropriate feasibility data to the sponsor and investigators. The TOC will oversee monitoring of the trial data and make recommendations to the TMG on whether there are any ethical or safety reasons as to why the trial should not continue or whether it needs to be modified. To this end, data on safety will be supplied to the TOC during the trial and all reports will be supplied in confidence.

### Data management

Electronic CRF, with programmed range checks, will be entered online via a secure REDCap database administered by BCTU. Authorised staff require an individual secure login username and password to access online data entry. All missing and ambiguous data are queried through the online system. The security of the System is governed by the policies of the University of Birmingham (Data Protection Registration number Z6195856), and each study site has arrangements in place for secure storage and processing of study data, in compliance with the University of Birmingham policies. The University carries appropriate Data Protection Registration coverage.

### Audit

BCTU staff will monitor electronic CRF returns for data completion and will work closely with site delivery teams to ensure that informed consent forms (ICFs) and CRFs are returned in a timely manner. The BCTU team will also cross reference ICF and CRF content against the trial protocol to ensure compliance with protocol standards and consistency in and completeness of data reporting.

## Ethics and dissemination

The trial will be conducted in accordance with the UK Policy Framework for Health and Social Care Research and applicable UK Acts of Parliament and Statutory Instruments (and relevant subsequent amendments), which include the Data Protection Act 2018 and the Principles of GCP as set out in the UK Statutory Instrument (2004/1031; and subsequent amendments). The protocol has been submitted to and approved by West Midlands Black Country Research Ethics Committee (reference number 22/WM/0214) and the Health Research Authority on 5 December 2022. Each trial site will confirm local NHS Trust Research and Development department capacity and capability prior to commencing recruitment. All participants will provide written informed consent prior to participation in the trial. University of Birmingham is the sponsor for the trial.

The findings from the pilot trial will be used to inform the design of a full-scale multicentre RCT. Outputs from the trial will be submitted for publication in peer-reviewed journals, presented at scientific conferences and disseminated to the public via patient charities/patient advocacy groups.

The manuscript will be prepared by the chief investigator and authorship will be determined by the trial publication policy. Any secondary publications and presentations prepared by investigators must be reviewed and approved by the TMG. Manuscripts must be submitted to the TMG in a timely fashion and in advance of being submitted for publication, to allow time for review and resolution of any outstanding issues. Authors must acknowledge that the trial was performed with the support of the sponsor (University of Birmingham) and BCTU. Intellectual property rights will be addressed in the Clinical Study Site Agreement between Sponsor and site.

### Patient and public involvement

Members of a local hospital patient charity which comprised people living with heart disease were initially involved providing constructive feedback regarding the research question and study design. A patient advisory group (PAG) was established to inform the development, testing and refinement of the APRAISE-AS app via a process of participatory design. The PAG supported user testing of the app and their input guided the frequency of reporting for the symptoms, timed up and go and health-related quality of life assessments to ensure acceptability in terms of burden from a patient’s perspective. The PAG also reviewed all patient facing materials and a member of the PAG also sits on the TMG. Our PPIE coapplicant will support dissemination of trial findings among relevant patient groups.

## Supplementary Material

Reviewer comments

Author's manuscript
