# Supplementary material for: Pilot study to evaluate the use of remote patient monitoring to guide the timing of valve intervention in patients with severe asymptomatic aortic stenosis (APRAISE-AS): study protocol for a randomised controlled trial delivered in two tertiary cardiac centres in the UK
Source: BMJ Open. 2024 Jun 10;14(6):e086587. doi: 10.1136/bmjopen-2024-086587 (PMC11168151; doi:10.1136/bmjopen-2024-086587)
Supplement: Author's manuscript [file bmjopen-2024-086587.draft_revisions.pdf]

BMJ Open is committed to open peer review. As part of this commitment we make the peer review history of every article we publish publicly available.

When an article is published we post the peer reviewers' comments and the authors' responses online. We also post the versions of the paper that were used during peer review. These are the versions that the peer review comments apply to.

The versions of the paper that follow are the versions that were submitted during the peer review process. They are not the versions of record or the final published versions. They should not be cited or distributed as the published version of this manuscript.

BMJ Open is an open access journal and the full, final, typeset and author-corrected version of record of the manuscript is available on our site with no access controls, subscription charges or pay-per-view fees (http://bmjopen.bmj.com).

If you have any questions on BMJ Open's open peer review process please email info.bmjopen@bmj.com

BMJ Open

APRAISE-AS Pilot Trial Protocol [Manuscript]

Journal:	*BMJ Open*
Manuscript ID	bmjopen-2024-086587
Article Type:	Protocol
Date Submitted by the Author:	18-Mar-2024
Complete List of Authors:	Khan, Nazish; University Hospitals Birmingham NHS Foundation Trust, Department of Cardiology (QEHB)<br>Steeds, Richard; Queen Elizabeth Hospital, Department of Cardiology<br>Kyte, Derek; University of Worcester,<br>Fabritz, Larissa; University of Birmingham, Institute of Cardiovascular Sciences<br>Collis, Philip; University of Birmingham, Centre for Patient Reported Outcomes Research and Institute of Applied Health Research<br>Chua, Winnie; The University of Birmingham, Institute of Cardiovascular Sciences<br>Stubbs, Clive; University of Birmingham<br>Mehta, Samir; University of Birmingham, Birmingham Clinical Trials Unit<br>Sun, Yongzhong; University of Birmingham, Birmingham Clinical Trials Unit (BCTU)<br>Nulty, Mary; University of Birmingham, Birmingham Clinical Trials Unit<br>Kirkham, Katie; University of Birmingham, Birmingham Clinical Trials Unit<br>Cotton, James; The Royal Wolverhampton Hospitals, Department of Cardiology
Keywords:	Patient Reported Outcome Measures, Valvular heart disease < CARDIOLOGY, Patient-Centered Care, Patient Satisfaction

SCHOLARONE™
Manuscripts

APRAISE-AS Pilot Trial Protocol [Manuscript]

A pilot study to assess the safety, effectiveness and practical utility of remote patient monitoring to guide the timing of valve intervention in patients with asymptomatic severe aortic stenosis: The APRAISE-AS pilot trial protocol

Contributors: Nazish Khan[1,2], Richard P. Steeds[1,2], Derek Kyte[3,4], Larissa Fabritz[1,5], Phil Collis[2], Winnie Chua[1], Clive Stubbs[6] Samir Mehta[6], Yongzhong Sun[6], Mary Nulty[6], Katie Kirkham[6], James M. Cotton[7,8]

1. Department of Cardiology (QEHB), University Hospitals Birmingham NHS Foundation Trust, UK
2. Institute of Cardiovascular Sciences, University of Birmingham, Birmingham, UK
3. School of Allied Health and Community, University of Worcester, Worcester, UK
4. Centre for Patient Reported Outcomes Research, Institute for Applied Health Research, University of Birmingham, Birmingham, UK
5. University Center of Cardiovascular Sciences & Department of Cardiology, University Heart and Vascular Center, University Hospital Hamburg Eppendorf, Hamburg, Germany
6. Birmingham Clinical Trials Unit, Institute for Applied Health Research, University of Birmingham, Birmingham, UK
7. Department of Cardiology, The Royal Wolverhampton Hospitals NHS Trust

Corresponding author: Nazish Khan E-mail: nazish.khan@nhs.net

Word count (including abstract, excluding references: 4,472)

Abstract

Introduction: Aortic stenosis (AS) is common affecting >13% of adults over the age of 75 years. In people who develop symptoms, without valve replacement, prognosis is dismal with mortality as high as 50% at 1 year. In asymptomatic patients, the timing of valve intervention is less well defined and a strategy of watchful waiting is recommended. Many, however, may develop symptoms and attribute this to age related decline, rather than worsening AS. Timely intervention in asymptomatic severe AS is critical, since delayed intervention often results in poor outcomes. Proactive surveillance of symptoms, quality of life and functional capacity should enable timely identification of people who will benefit from aortic valve replacement. There are no data however, to support the clinical and cost effectiveness of such an approach in a healthcare setting in the United Kingdom. The aim of this pilot

For peer review only

trial is to test the feasibility of a full-scale randomised controlled trial to determine the utility of proactive surveillance in people with asymptomatic severe AS to guide the timing of intervention.

Methods and analysis: APRAISE-AS is a multi-centre, non-blinded, two-arm, parallel group randomised controlled trial of up to 66 participants aged >18 years with asymptomatic severe AS. Participants will be randomised to either standard care or standard care supplemented with the APRAISE-AS intervention. Primary outcomes will capture; adherence to and participant acceptability of the intervention, recruitment and retention rates, and completeness of data collection. The findings will be used to inform the sample size and most appropriate outcome measure(s) for a full-scale RCT and health economic evaluation.

Ethics and dissemination: Ethical approval was granted by the Black Country REC, reference: 22/WM/0214. Results will be submitted for publication in peer reviewed journals and disseminated at local, regional and national meetings where appropriate.

Trial registration number(s): ISRCTN reference: 19413194 prospectively registered 14.07.2023.

Strengths and limitations of this study:

- The study uses a randomised controlled trial design to assess the feasibility of how subjective (symptoms & health related quality of life) and objective (functional capacity) patient reported data might supplement standard care to inform the timeliness of aortic valve replacement.
- The intervention has been developed via a process of participatory design in collaboration with patients and clinicians.
- The intervention provides an innovative way of using digital technology in patients with heart valve disease (aortic stenosis), to support patient engagement and improve the patient pathway through facilitating enhanced remote patient monitoring, near real time clinician assessment and risk stratification to inform the optimum time at which valve intervention should take place.
- The study is not powered to determine clinical outcomes but is designed to determine the feasibility of conducting a full-scale RCT to understand the clinical utility and cost-effectiveness of integrating an innovative approach to proactive, remote patient monitoring into the clinical management pathway of people with asymptomatic severe AS.

Key words: asymptomatic severe aortic stenosis, valve replacement, symptoms, patient reported outcome measures, functional capacity

For peer review only

INTRODUCTION

Heart valve disease (HVD) affects 1.5 million people over the age of 65 years in the United Kingdom and is an important cause of morbidity and mortality (1). Aortic stenosis (AS) is the most common acquired form of HVD encountered in clinical practice (2). The overall burden of disease in the general population is substantial with an incidence as high as 13% in those aged ≥ 75 years (3). Prevalence and associated healthcare costs secondary to AS are likely to rise in view of an increasingly ageing, frail and multi-morbid population.

AS is characterised by insidious thickening and calcification of a native aortic valve and is an active process driven by lipid accumulation, inflammation and fibrosis (4, 5). Whilst the underlying pathophysiology of the condition is similar to the process of atherosclerosis, there are no proven medical treatments that can limit disease progression and AS is treated by aortic valve replacement (AVR) (2).

Severity of AS is determined by echocardiographic parameters including the maximal velocity, mean pressure gradient and aortic valve area (6). Intervention is mandated in severe AS following the development of symptoms including breathlessness, dizziness, blackouts, chest pain and palpitations. Prognosis is dismal following the development of symptoms, with 1-year mortality as high as 50%, and prompt intervention is therefore critical (7) (6).

In asymptomatic patients however, the timing of valve intervention remains less well defined. A strategy of watchful waiting is adopted, and patients are counselled on the importance of immediate reporting should symptoms develop. Regular clinical surveillance is mandatory and a full physical assessment and echocardiogram should be undertaken at least every six months (6). The slowly progressive nature of AS combined with the advanced age of the affected population, however, predispose to underreporting and/or underestimation of symptoms (8). Risks associated with a watchful waiting approach include irreversible myocardial damage, sudden cardiac death (with an incidence of 1.5% per year), delayed symptom reporting and dying whilst awaiting valvular intervention (9, 10).

Unmet need

The optimal timing of valve intervention in asymptomatic severe AS is currently under investigation in a number of randomised controlled clinical trials; the outcomes of EARLY-TAVR and EVOLVED, will

For peer review only

provide insight into whether proactive surveillance, diagnostic imaging and an understanding of the optimum window of opportunity can be implemented in clinical practice to guide the timing of intervention in patients with asymptomatic severe AS (11, 12).

Objective

To date there have been no high-quality, multicentre RCTs to test the clinical and cost-effectiveness of whether proactive symptom surveillance and remote patient monitoring using a software application (“app”) in addition to a traditional “watchful waiting” strategy in adult patients with asymptomatic severe AS allows for earlier identification of symptoms and timelier aortic valve intervention leading to improved clinical outcomes. However, before a large RCT can be undertaken, a pilot trial is required to inform the most appropriate outcome measures and sample size for a full-scale RCT. Therefore the APRAISE-AS study will pilot the trial protocol, in order to assess the feasibility and potential refinement of the trial design and/or trial intervention prior to undertaking a larger randomised controlled trial investigating whether proactive reporting of symptoms, definitive disease specific patient reported outcome measures (PROMs) and assessment of frailty in addition to standard care can be used to help inform the timeliness of valve intervention in patients with asymptomatic severe aortic stenosis.

METHODS

Trial design and setting

This protocol adheres to guidance from the Standard Protocol Items: Recommendations for Interventional Trials (SPIRIT) and a SPIRIT checklist is included in an accompanying supplementary file (13, 14). The APRAISE-AS pilot trial is a multi-centre, non-blinded, two-arm, parallel group randomised controlled pilot trial of up to 66 participants aged 18 years or over with asymptomatic severe AS. The pilot trial will be conducted in two tertiary cardiology and cardiothoracic surgery Heart Valve centres in the West Midlands. Participants will be randomised in a 1:1 ratio to receive either standard care or standard care supplemented with the APRAISE-AS intervention. The study trial schema is described in figure 1.

Recruitment and eligibility

Recruitment commenced in February 2023 in The Royal Wolverhampton NHS Trust and October 2023 at the Queen Elizabeth Hospital Birmingham and closed in January 2024 and February 2024 respectively. Patients were invited to participate if they had a diagnosis of asymptomatic severe AS and if they met the following eligibility criteria:

For peer review only

Inclusion criteria

- Patients diagnosed with asymptomatic severe AS of a native valve, in line with current international guideline and defined as either:
  - **(a)** Aortic valve area <1cm^2 and mean pressure gradient >40mmHg and LVEF >50% (in the last 6 months), OR
  - **(b)** Aortic valve area <1cm^2 and mean pressure gradient ≤ 40mmHg, stroke volume index ≤ 35ml/m^2 and LVEF >50% (in the last 6 months)
- Able to give written informed consent
- Age ≥ 18 years
- Able to access and use app-based programmes

Exclusion criteria

- Lack of capacity to understand the nature of the study and possible consequences as determined by their attending clinician
- Moderate or more severe disease of other valves e.g. mitral stenosis/regurgitation
- Co-morbidities that in the opinion of the cardiac surgeon/cardiologist preclude successful valve intervention e.g. life expectancy of less than 2 years

Participation identification, recruitment and informed consent

Adult patients with a confirmed diagnosis of severe asymptomatic AS of a native valve were recruited. Patients were identified for eligibility and approached for recruitment via one of three routes: (i) following automated e-mail alert for people who meet echocardiographic parameters associated with severe AS, (ii) new referrals seen in out-patient clinics or following identification at ward level, or (iii) from cardiac investigation department echocardiogram diagnostic lists. Existing patients known to the respective cardiac research teams were also be approached for recruitment. Following confirmation of eligibility by the cardiovascular research team (clinicians/pharmacists/nurses), potential participants were contacted by telephone to gauge interest in the trial and request permission for a participant information sheet to be sent to them either via e-mail or by post. In addition, potential participants were also be given a participant information sheet during their clinic appointment. The research team gave potential participants sufficient time to review the information provided, discuss the trial with family members and/or their GP. The Chief Investigator/site Principal Investigators ensured that participants fully understand the aim of the trial, the intervention and potential benefits and drawbacks associated with participation. On receiving an expression of interest

For peer review only

from the potential participant, they were invited to an outpatient appointment where informed consent for participation was obtained in addition to completion of baseline assessments of symptom status, functional capacity and quality of life were undertaken prior to randomisation. Participants were reminded that participation is voluntary and they may withdraw from the trial at any time and it will not affect the care they receive.

Randomisation

Following baseline assessments, participants were randomised at the level of the individual in a 1:1 ratio to either standard care (control arm) or standard care supplemented with the APRAISE-AS intervention (intervention arm). Randomisation was undertaken by Birmingham Clinical Trials Unit (BCTU) using a computer-generated sequence created by a programmer independent to the study team. A minimisation algorithm has been used within the randomisation system to ensure balance in the intervention allocations over the following variables:

- Age ('< 50 years' and '≥ 50 years')
- Sex ('male' and 'female')
- Ethnicity ('white' and 'non-white')

To avoid the possibility of the intervention allocation becoming predictable, a random element will be included in the algorithm (unspecified here), so that each participant has a chance of being randomised to the opposite intervention that they would have otherwise received.

Interventions

Participants assigned to standard care (control arm) will continue to receive usual care which includes a 6 monthly valve clinical review and surveillance echocardiogram (as part of a watchful waiting strategy). For participants assigned to the intervention arm, standard care will be supplemented with the APRAISE-AS intervention. The APRAISE-AS intervention includes an app and a clinician's portal which has been developed via a process of participatory design in collaboration with our patients and clinicians, which will collect both subjective and objective data on a patients' health and well-being following their diagnosis of asymptomatic severe AS. This information will be presented to the healthcare team via a bespoke platform which allows near real-time surveillance of the patients' healthcare data (Clinician's Portal). The aim of the app is to facilitate clinical prioritisation of patients who demonstrate symptoms or early signs of decompensation to allow for escalation of treatment and/or earlier intervention. These self-reported patient tests will be used to supplement routine clinical appointments.

For peer review only

For participants randomised to standard care plus the APRAISE-AS intervention, a member of the research team will install and provide access to the app on the participant's own, or a Trust sourced, device. Over a 12-month period, participants will use the app to self-report information relating to:

- Symptoms [(assessed using New York Heart Association and Canadian Cardiovascular System classification) dyspnoea/fatigue/palpitations/angina and syncope] on a weekly basis.
- Frailty/functional capacity (Timed Up and Go test) every two weeks [frailty, assessed using gait speed, is known to correlate with major morbidity and mortality after valve surgery and provides a continuous reproducible measure allowing for an assessment of functional status over time (15-17)].
- Disease specific PROMs using the Kansas City Cardiomyopathy Questionnaire (KCCQ) once a month [KCCQ has been studied extensively in the setting of AS and has been found to be a reliable, reproducible and valid measure of symptoms, functional status, quality of life and prognosis (18)].

Participants will receive automated reminders prior to submitting a self-assessment via the app and 24 hours after a failure to submit their report, if necessary. Severe symptoms noted by the patient will trigger an email alert to the APRAISE-AS clinical team (dedicated monitored email inbox) and a simultaneous patient notification advising them to make contact with their clinical team during office hours (or to use standard NHS support mechanisms outside of these hours). The clinical team will monitor for notifications and respond in line with standard care recommendations during office hours. Actions taken in response to an alert will be logged in the patient's electronic healthcare record by a member of the APRAISE-AS clinical team (including members of the cardiovascular research team).

Outcome measures and study procedures

The primary aim of this study is to pilot the trial protocol and assess the feasibility and potential refinement of the trial design prior to undertaking a larger randomised controlled trial (RCT) investigating whether proactive surveillance/reporting of symptoms, definitive disease specific PROMs and assessment of frailty in addition to standard care can be used to help inform the timeliness of valve intervention in patients with asymptomatic severe AS. In addition, the pilot will allow for refinement of the trial design and/or trial intervention prior to undertaking a larger multi-centre RCT which will assess the clinical and cost-effectiveness of remote patient monitoring in this setting. The aim of using the outcomes to enable pathway transformation and service redesign through the incorporation of remote monitoring into the clinical management pathway for people with asymptomatic severe AS.

For peer review only

The pilot trial will:

- Test and pilot the trial protocol (including recruitment and retention rates, data collection processes, data completeness and adherence to the APRAISE-AS intervention);
- Inform selection of the most appropriate primary outcome measure for a full-scale multicentre RCT;
- Provide data to help estimate the sample size for a full-scale multicentre RCT;
- Provide a platform to develop and pilot the processes to capture costs and outcomes to inform the health economic evaluation for a full-scale multicentre RCT;
- Determine key participation criteria for centre involvement in a full-scale RCT.

Primary outcomes

In addition to piloting the trial protocol, the pilot study will assess:

- The utility of the “app” using quantitative (e.g. usage data, data completeness) and qualitative (interviews) data
- Engagement with and acceptability of the intervention
- Recruitment rate (per month)
- Number of withdrawals and losses to follow-up (with reasons)

All study staff and participants will be invited to complete a trial process questionnaire at the end of the study, which will evaluate aspects surrounding:

- The extent and frequency of data collection
- Randomisation procedure
- Acceptability of the intervention (e.g. for patients using the mobile app rating score (MARS) tool.
- Appropriateness of the frequency of APRAISE-AS reporting, alert thresholds and subsequent management.
- The utility of the clinician’s portal and embedding remote monitoring into the clinical management pathway.

Study Procedures

During the baseline visit participants’ demographic (age, gender and ethnicity) and biomarker (creatinine, creatinine clearance) data will be collected. Baseline assessment of symptoms, functional capacity (using the Timed Up and Go test) and health related quality of life (using the disease specific

For peer review only

KCCQ) will be undertaken. Undertaking an objective assessment of frailty using a parameter such as gait speed, which correlates with major morbidity and mortality after valve surgery, provides a continuous reproducible measure allowing for an assessment of functional status over time (15-17). In addition, a comprehensive determination of health-related quality of life for patients undergoing valvular intervention, incorporating heart failure specific measures is recommended (15). The disease specific patient reported outcome measure (PROM) Kansas City Cardiomyopathy Questionnaire (KCCQ) has been studied extensively in the setting of AS and has been found to be a reliable, reproducible and valid measure of symptoms, functional status, quality of life and prognosis (18).

As described in table 1, all participants will complete the timed up and go test and paper versions of the KCCQ and symptom tracker questionnaires (in clinic) at baseline (prior to randomisation), and when attending the two subsequent follow up visits. For participants assigned to the standard care arm, post-randomisation timed up and go, symptom and health status questionnaires will only be completed at the routine 6- and 12-months follow-up appointments. For those participants assigned to the intervention arm, this information will also be collected via the app at pre-specified frequencies post-randomisation as well as during their routine 6- and 12-month follow-up appointments.

Adherence with the APRAISE-AS intervention will be assessed by calculating the number and percentage (with 95% CI) of patients who complete the symptom surveillance, frailty and quality of life assessments as scheduled. To be considered adherent, patients will need to have submitted their report within 24 hours of the scheduled time-point. Incomplete submissions (i.e. with some questions not answered) will be accepted for the purpose of measuring adherence. Ad-hoc reports (i.e. those completed outside the scheduled reporting periods) will not contribute to the assessment of adherence, although we will assess the number of APRAISE-AS reports completed by each patient.

Sample size and justification

Since this is a pilot study a formal sample size calculation has not been performed. In order to obtain estimates of the parameters needed for sample size determination, in line with published recommendations for pilot studies, 30 patients are required (19, 20). Assuming a loss to follow-up rate of 10% over the 12-month follow up period, the study will require 33 patients to be randomised to each group, with a total of 66 patients overall. This will also allow for estimation of recruitment and retention rates with 95% CI maximum widths of 20% and 25%, respectively.

Analysis of outcome measures

For peer review only

A separate Statistical Analysis Plan (SAP) will be produced and will provide a more comprehensive description of the planned statistical analyses. A brief outline of the planned analyses is given below. The primary comparison groups will be composed of those randomised to standard care supplemented with the APRAISE-AS intervention (intervention group) versus those randomised to standard care (control group). All analyses will be based on the intention to treat (ITT) principle, i.e. all patients will be analysed in the treatment group to which they were randomised irrespective of compliance with the allocated treatment or other protocol deviation. The data analysis for this pilot trial will mainly be descriptive and focus on confidence interval estimation, with no hypothesis testing performed. The reporting and presentation of this trial will be in accordance with the CONSORT guidelines for randomised trials.

The pilot data will also help inform the selection of the most appropriate primary outcome measure for the main RCT and provide contributory data to facilitate estimation of the sample size required for the main RCT. Analysis methods will be chosen according to the data type of the outcome under investigation, in brief:

- *Continuous outcomes (e.g. quality of life)*: These data will be summarised using means and standard deviations, with differences in means with 95% confidence intervals reported. Longitudinal plots of the data over time will also be constructed for visual presentation of the data.

- *Categorical (dichotomous) outcomes (e.g. hospitalisation rates)*: The number of patients and percentages experiencing the event will be summarised between groups.
- *Time to Event outcomes (e.g. time to symptom development, valve replacement, mortality):* The numbers of patients and percentages experiencing the event will be summarised over time between groups. Kaplan-Meier survival curves will be constructed for visual presentation of time-to-event data.

Missing data and sensitivity analyses

Every attempt will be made to collect full follow-up data on all study patients; it is thus anticipated that missing data will be minimal. The assessment of missing data is an outcome measure of this pilot trial and forms part of the assessment of the feasibility of a future trial. The amount of missing data at outcome measure level will also be considered when assessing and identifying a suitable primary outcome for the future trial. As this is a pilot trial, no formal sensitivity analysis will be conducted.

Planned interim analysis

For peer review only

Since this is a pilot study a planned interim analysis will not be undertaken. However, the trial oversight committee will access to recruitment, retention and data collection information.

Planned final analysis

The primary analysis for the study will occur once all patients have completed their 12-month assessment and corresponding outcome data have been entered onto the study database (REDCap) and validated as being ready for analysis. This analysis will include data items up to and including the 12-month assessment and no further.

Health Economics

A health economic evaluation is not planned for this pilot trial. However, data will be collected to capture costs and outcomes to inform the health economic evaluation for a multi-centre full-scale RCT. Data will be collated in relation to cardiology clinical staff time and activity in response to APRAISE-AS notifications, including ad-hoc telephone consultations, in-patient's hospitalisations and onward referrals for consideration of aortic valve replacement, in addition to NHS costs attributable to maintenance of the APRAISE-AS clinician's portal. Where possible data on healthcare resource utilisation will be collected from both electronic patient records and case report forms either during study follow up visits or following response to a severe symptom alert.

Monitoring, trial management and oversight

The Chief Investigator has overall responsibility for the study. The Trial Management Group (TMG) consisting of the cardiovascular research team (including chief investigator and site principal investigators) will oversee day to day running of trial. Due to the small sample size and pilot nature of this trial, the Trial Steering and Data Monitoring Committees have been combined into a single Trial Oversight Committee (TOC). The TOC will provide overall oversight of the trial, including the practical aspects of the study, as well as ensuring that the study is run in a way which is both safe for the patients and provides appropriate feasibility data to the sponsor and investigators. The TOC will oversee monitoring of the trial data and make recommendations to the TMG on whether there are any ethical or safety reasons as to why the trial should not continue or whether it needs to be modified. To this end, data on safety will be supplied to the TOC during the trial and all reports will be supplied in confidence.

Data management

Electronic case report forms, with programmed range checks, will be entered online via a secure REDCap database administered by BCTU. Authorised staff require an individual secure login username

For peer review only

and password to access online data entry. All missing and ambiguous data are queried through the online system. The security of the System is governed by the policies of the University of Birmingham (Data Protection Registration number Z6195856), and each study site has arrangements in place for secure storage and processing of study data, in compliance with the University of Birmingham policies. The University carries appropriate Data Protection Registration coverage.

Audit

BCTU staff will monitor electronic case report forms (CRF) returns for data completion and will work closely with site delivery teams to ensure that informed consent forms (ICFs) and CRFs are returned in a timely manner. The BCTU team will also cross reference ICF and CRF content against the trial protocol to ensure compliance with protocol standards and consistency in and completeness of data reporting.

ETHICS AND DISSEMMINATION

The trial will be conducted in accordance with the UK Policy Framework for Health and Social Care Research and applicable UK Acts of Parliament and Statutory Instruments (and relevant subsequent amendments), which include the Data Protection Act 2018 and the Principles of GCP as set out in the UK Statutory Instrument (2004/1031; and subsequent amendments). The protocol has been submitted to and approved by West Midlands Black Country Research Ethics Committee (reference number 22/WM/0214) and the Health Research Authority on 05/12/2022. Each trial site will confirm local NHS Trust Research & Development department capacity and capability prior to commencing recruitment. All participants will provide written informed consent prior to participation in the trial. University of Birmingham are the sponsor for the trial.

The findings from the pilot trial will be used to inform the design of a full-scale multicentre RCT. Outputs from the trial will be submitted for publication in peer reviewed journals, presented at scientific conferences and disseminated to the public via patient charities/patient advocacy groups. The manuscript will be prepared by the Chief Investigator and authorship will be determined by the trial publication policy. Any secondary publications and presentations prepared by Investigators must be reviewed and approved by the TMG. Manuscripts must be submitted to the TMG in a timely fashion and in advance of being submitted for publication, to allow time for review and resolution of any outstanding issues. Authors must acknowledge that the trial was performed with the support of the sponsor (University of Birmingham) and BCTU. Intellectual property rights will be addressed in the Clinical Study Site Agreement between Sponsor and site.

For peer review only

Patient and Public Involvement

Members of a local hospital patient charity which comprised people living with heart disease were initially involved providing constructive feedback regarding the research question and study design. A patient advisory group (PAG) was established to inform the development, testing and refinement of the APRAISE-AS app via a process of participatory design. The PAG supported user testing of the app and their input guided the frequency of reporting for the symptoms, timed up and go and health related quality of life assessments to ensure acceptability in terms of burden from a patient's perspective. The PAG also reviewed all patient facing materials and a member of the PAG also sits on the TMG. Our PPIE co-applicant will support dissemination of trial findings among relevant patient groups.

Author contributions:

NK is the Chief Investigator and takes final responsibility for the study design, conduct and decision to submit for publication. NK prepared the first draft of the protocol with approval from all authors. JC is co-Chief Investigator and site Principal Investigator, RS is a site Principal Investigator, both provided clinical expertise. CS, MN, KK provided trials expertise. SM (statistician) designed and wrote the statistical analysis plan. DK, LF, WC provided expertise in terms of PROMs trial methodology and the delivery of remote monitoring in the context of a clinical trial. PC provided expertise in terms of PROMs and PPIE. All investigators provided advice and critical input on the study design. All authors critically revised the manuscript for its important intellectual content. All authors read and approved the final manuscript.

Funding statement

This study is funded by the National Institute for Health and Care Research (NIHR) under its Research for Patient Benefit (RfPB) Programme (Grant Reference Number NIHR202810) and sponsored by the University of Birmingham (reference: RG_22-094).

Disclaimer

The views expressed are those of the author(s) and not necessarily those of the NIHR or the Department of Health and Social Care.

NK and JC were also financially supported by the West Midlands Clinical Research Network and Wolverhampton Coronary Care After Support Patient Group. The views expressed are those of the author(s) and not necessarily those of the NIHR or the Department of Health and Social Care.

For peer review only

Competing interests' statement:

LF reports grants from the BHF (AA/18/2/34218), EU Horizon 2020 CATCH ME (grant agreement number 633196), MAESTRIA (grant agreement number 965286), AFFECT-EU (grant agreement number 847770, NIHR award 1002898 (APRAISE-AS) at University of Birmingham, and German Center for Cardiovascular Research (DZHK). LF has also received institutional research grants from governmental funding agencies, charities and companies in biomedical research. LF is part of the scientific advisory board of a patient initiative.

DK reports grants from the NIHR and Kidney Research UK outside the submitted work. NK reports grants from the NIHR.

Patient consent

Not required

References:

1. Voice HV. Heart Valve Voice https://heartvalvevoice.com2019 [Available from: https://heartvalvevoice.com/heart-valve-disease/prevalence.

2. Osnabrugge RL, Mylotte D, Head SJ, Van Mieghem NM, Nkomo VT, LeReun CM, et al. Aortic stenosis in the elderly: disease prevalence and number of candidates for transcatheter aortic valve replacement: a meta-analysis and modeling study. Journal of the American College of Cardiology. 2013;62(11):1002-12.

3. Kwok CS, Bagur R, Rashid M, Lavi R, Cibelli M, de Belder MA, et al. Aortic stenosis and non-cardiac surgery: A systematic review and meta-analysis. International journal of cardiology. 2017;240:145-53.

4. Otto CM. Valvular aortic stenosis: disease severity and timing of intervention. Journal of the American College of Cardiology. 2006;47(11):2141-51.

5. Everett RJ, Clavel MA, Pibarot P, Dweck MR. Timing of intervention in aortic stenosis: a review of current and future strategies. Heart. 2018;104(24):2067-76.

6. Vahanian A, Beyersdorf F, Praz F, Milojevic M, Baldus S, Bauersachs J, et al. 2021 ESC/EACTS Guidelines for the management of valvular heart disease. Eur J Cardiothorac Surg. 2021;60(4):727-800.

7. Leon MB, Smith CR, Mack M, Miller DC, Moses JW, Svensson LG, et al. Transcatheter aortic-valve implantation for aortic stenosis in patients who cannot undergo surgery. N Engl J Med. 2010;363(17):1597-607.

8. Tribouilloy C, Rusinaru D, Bohbot Y, Marechaux S, Vanoverschelde JL, Enriquez-Sarano M. How Should Very Severe Aortic Stenosis Be Defined in Asymptomatic Individuals? J Am Heart Assoc. 2019;8(3):e011724.

9. Rosenhek R, Zilberszac R, Schemper M, Czerny M, Mundigler G, Graf S, et al. Natural history of very severe aortic stenosis. Circulation. 2010;121(1):151-6.

10. Taniguchi T, Morimoto T, Shiomi H, Ando K, Kanamori N, Murata K, et al. Initial Surgical Versus Conservative Strategies in Patients With Asymptomatic Severe Aortic Stenosis. Journal of the American College of Cardiology. 2015;66(25):2827-38.

For peer review only

11. Généreux P. Evaluation of Transcatheter Aortic Valve Replacement Compared to SurveilLance for Patients With AsYmptomatic Severe Aortic Stenosis (EARLY TAVR) ClinicalTrials.gov: ClinicalTrials.gov; 2019 [Available from: https://clinicaltrials.gov/ct2/show/NCT03042104.
12. Dweck M. Early Valve Replacement Guided by Biomarkers of LV Decompensation in Asymptomatic Patients With Severe AS (EVoLVeD) ClinicalTrials.gov2019 [Available from: https://clinicaltrials.gov/ct2/show/NCT03094143.
13. Chan AW, Tetzlaff JM, Gotzsche PC, Altman DG, Mann H, Berlin JA, et al. SPIRIT 2013 explanation and elaboration: guidance for protocols of clinical trials. BMJ. 2013;346:e7586.
14. Chan AW, Tetzlaff JM, Altman DG, Laupacis A, Gotzsche PC, Krleza-Jeric K, et al. SPIRIT 2013 statement: defining standard protocol items for clinical trials. Ann Intern Med. 2013;158(3):200-7.
15. Kappetein AP, Head SJ, Genereux P, Piazza N, van Mieghem NM, Blackstone EH, et al. Updated standardized endpoint definitions for transcatheter aortic valve implantation: the Valve Academic Research Consortium-2 consensus document. European heart journal. 2012;33(19):2403-18.
16. Afilalo J, Lauck S, Kim DH, Lefevre T, Piazza N, Lachapelle K, et al. Frailty in Older Adults Undergoing Aortic Valve Replacement: The FRAILTY-AVR Study. Journal of the American College of Cardiology. 2017;70(6):689-700.
17. Sepehri A, Beggs T, Hassan A, Rigatto C, Shaw-Daigle C, Tangri N, et al. The impact of frailty on outcomes after cardiac surgery: a systematic review. J Thorac Cardiovasc Surg. 2014;148(6):3110-7.
18. Arnold SV, O'Brien SM, Vemulapalli S, Cohen DJ, Stebbins A, Brennan JM, et al. Inclusion of Functional Status Measures in the Risk Adjustment of 30-Day Mortality After Transcatheter Aortic Valve Replacement: A Report From the Society of Thoracic Surgeons/American College of Cardiology TVT Registry. JACC Cardiovasc Interv. 2018;11(6):581-9.
19. Lancaster GA, Dodd S, Williamson PR. Design and analysis of pilot studies: recommendations for good practice. J Eval Clin Pract. 2004;10(2):307-12.
20. Browne RH. On the use of a pilot sample for sample size determination. Stat Med. 1995;14(17):1933-40.

For peer review only

APRAISE-AS Pilot Trial Protocol [Figures & Table]

A pilot study to assess the safety, effectiveness and practical utility of remote patient monitoring to guide the timing of valve intervention in patients with asymptomatic severe aortic stenosis: The APRAISE-AS pilot trial protocol

Contributors: Nazish Khan[1,2], Richard P. Steeds[1,2], Derek Kyte[3,4], Larissa Fabritz[1,5], Phil Collis[2], Winnie Chua[1], Clive Stubbs[6] Samir Mehta[6], Yongzhong Sun[6], Mary Nulty[6], Katie Kirkham[6], James M. Cotton[7,8]

1. Department of Cardiology (QEHB), University Hospitals Birmingham NHS Foundation Trust, UK
2. Institute of Cardiovascular Sciences, University of Birmingham, Birmingham, UK
3. School of Allied Health and Community, University of Worcester, Worcester, UK
4. Centre for Patient Reported Outcomes Research, Institute for Applied Health Research, University of Birmingham, Birmingham, UK
5. University Center of Cardiovascular Sciences & Department of Cardiology, University Heart and Vascular Center, University Hospital Hamburg Eppendorf, Hamburg, Germany
6. Birmingham Clinical Trials Unit, Institute for Applied Health Research, University of Birmingham, Birmingham, UK
7. Department of Cardiology, The Royal Wolverhampton Hospitals NHS Trust

Corresponding author: Nazish Khan E-mail: nazish.khan@nhs.net

For peer review only

Figure 1: Trial schema/flow chart

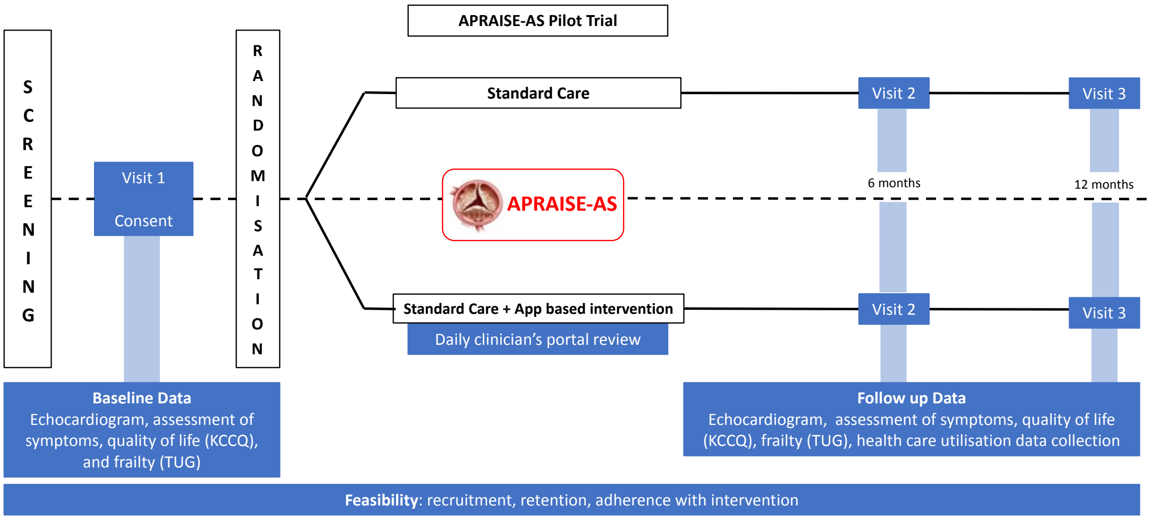

Inclusion Criteria

- Patients diagnosed with asymptomatic sAS of a native valve, with either:

(a) Aortic valve area <1cm2 and mean pressure gradient >40mmHg and LVEF >50% (in the last 6 months) OR
(b) Severe AS (low flow, low gradient): Aortic valve area <1cm2 and mean pressure gradient ≤ 40mmHg and LVEF >50% and SVi ≤ 35mL/m2 (in the last 6 months)

- Able to give written informed consent
- Age ≥18 years
- Able to access and use app-based programmes (using the English language)

Exclusion Criteria

- Lack of capacity to understand the nature of the study and possible consequences
- Significant disease of other valves e.g. mitral stenosis/regurgitation
- Co-morbidities that in the opinion of the cardiac surgeon/cardiologist preclude successful valve intervention e.g. life expectancy of less than 2 years

For peer review only

Table 1. Schedule of Assessments for KCCQ (PROMs), symptoms and Timed Up & Go (frailty) data capture

Assessment	Baseline/prior to randomisation	Once a week ± 2 days	Once every 2 weeks ± 2 days	Once a month ± 2 days	Month 6 ± 2 days	Month 12 ± 2 days	Ad-hoc reporting	Clinical site continuous daily monitoring (intervention arm)	Responding to severe symptoms* Clinical site continuous daily monitoring
Control Group									
Symptoms	x				x	x			x
Timed Up & Go	x				x	x			x
KCCQ	x				x	x			x
Intervention Group									
Symptoms	x	x			x	x	x	x	x
Timed Up & Go	x		x		x	x	x	x	x
KCCQ	x			x	x	x	x	x	x

*following submission of a severe symptom, the clinical team will document the action taken and outcome within the notes section in the clinician's portal and within the patient record used as per standard local clinical practice.

For peer review only

SPIRIT | CONSORT OUTCOMES Reporting Guidelines for Trial Outcomes

SPIRIT-Outcomes 2022 Checklist (for combined completion of SPIRIT 2013 and SPIRIT-Outcomes 2022 items)[a]

Section	Item No.	SPIRIT 2013 Item	SPIRIT-Outcomes 2022 item	Location Reported[b]
**Administrative information**				
Title	1	Descriptive title identifying the study design, population, interventions, and, if applicable, trial acronym	-	1
Trial registration	2a	Trial identifier and registry name. If not yet registered, name of intended registry	-	1
	2b	All items from the World Health Organization Trial Registration Data Set	-	n/a
Protocol version	3	Date and version identifier	-	1
Funding	4	Sources and types of financial, material, and other support	-	2
Roles and responsibilities	5a	Names, affiliations, and roles of protocol contributors	-	5 -8
	5b	Name and contact information for the trial sponsor	-	5
	5c	Role of study sponsor and funders, if any, in study design; collection, management, analysis, and interpretation of data; writing of the report; and the decision to submit the report for publication, including whether they will have ultimate authority over any of these activities	-	2, 46
	5d	Composition, roles, and responsibilities of the coordinating centre, steering committee, endpoint adjudication committee, data management team, and other individuals or groups overseeing the trial, if applicable (see Item 21a for data monitoring committee)	-	3,4, 8,9, 46
**Introduction**				
Background and rationale	6a	Description of research question and justification for undertaking the trial, including summary of relevant studies (published and unpublished) examining benefits and harms for each intervention	-	12, 20-22
	6b	Explanation for choice of comparators	-	22-23
Objectives	7	Specific objectives or hypotheses	-	12, 22-23

For peer review only - http://bmjopen.bmj.com/site/about/guidelines.xhtml

SPIRIT | CONSORT OUTCOMES
Reporting Guidelines for Trial Outcomes

Section	Item No.	SPIRIT 2013 Item	SPIRIT-Outcomes 2022 item	Location Reported[b]
Trial design	8	Description of trial design including type of trial (eg, parallel group, crossover, factorial, single group), allocation ratio, and framework (eg, superiority, equivalence, noninferiority, exploratory)	-	12 - 13, 14, 23 - 24
**Methods: Participants, interventions, and outcomes**				
Study setting	9	Description of study settings (eg, community clinic, academic hospital) and list of countries where data will be collected. Reference to where list of study sites can be obtained	-	12,
Eligibility criteria	10	Inclusion and exclusion criteria for participants. If applicable, eligibility criteria for study centres and individuals who will perform the interventions (eg, surgeons, psychotherapists)	-	24
Interventions	11a	Interventions for each group with sufficient detail to allow replication, including how and when they will be administered (for specific guidance see TIDieR checklist and guide)	-	12 - 13, 28
	11b	Criteria for discontinuing or modifying allocated interventions for a given trial participant (eg, drug dose change in response to harms, participant request, or improving/worsening disease)	-	29
	11c	Strategies to improve adherence to intervention protocols, and any procedures for monitoring adherence (eg, drug tablet return, laboratory tests)	-	n/a
	11d	Relevant concomitant care and interventions that are permitted or prohibited during the trial	-	24
Outcomes	12	Primary, secondary, and other outcomes, including the specific measurement variable (eg, systolic blood pressure), analysis metric (eg, change from baseline, final value, time to event), method of aggregation (eg, median, proportion), and time point for each outcome. Explanation of the clinical relevance of chosen efficacy and harm outcomes is strongly recommended	-	12 - 13, 29 - 30, 20

For peer review only - http://bmjopen.bmj.com/site/about/guidelines.xhtml

Section	Item No.	SPIRIT 2013 Item	SPIRIT-Outcomes 2022 item	Location Reported[b]
	12.1		Provide a rationale for the selection of the domain for the trial's primary outcome	22
	12.2		If the analysis metric for the primary outcome represents within-participant change, define and justify the minimal important change in individuals	n/a
	12.3		If the outcome data collected are continuous but will be analyzed as categorical (method of aggregation), specify the cutoff values to be used	n/a
	12.4		If outcome assessments will be performed at several time points after randomization, state the time points that will be used for analysis	29 - 34
	12.5		If a composite outcome is used, define all individual components of the composite outcome	n/a
Participant timeline	13	Time schedule of enrolment, interventions (including any run-ins and washouts), assessments, and visits for participants. A schematic diagram is highly recommended (see Figure)	-	32, 33
Sample size	14	Estimated number of participants needed to achieve study objectives and how it was determined, including clinical and statistical assumptions supporting any sample size calculations	-	43
	14.1		Define and justify the target difference between treatment groups (eg, the minimal important difference)	n/a
Recruitment	15	Strategies for achieving adequate participant enrolment to reach target sample size	-	12, 25 - 26
**Methods: Assignment of interventions (for controlled trials)**				
Allocation:				
Sequence generation	16a	Method of generating the allocation sequence (eg, computer-generated random numbers), and list of any factors for stratification. To reduce predictability of a random sequence, details of any planned restriction (eg, blocking) should be provided in a separate document that is unavailable to those who enrol participants or assign interventions	-	27

For peer review only - http://bmjopen.bmj.com/site/about/guidelines.xhtml

Section	Item No.	SPIRIT 2013 Item	SPIRIT-Outcomes 2022 item	Location Reported[b]
Allocation concealment mechanism	16b	Mechanism of implementing the allocation sequence (eg, central telephone; sequentially numbered, opaque, sealed envelopes), describing any steps to conceal the sequence until interventions are assigned	-	27 - 28
Implementation	16c	Who will generate the allocation sequence, who will enrol participants, and who will assign participants to interventions	-	27
Blinding (masking)	17a	Who will be blinded after assignment to interventions (eg, trial participants, care providers, outcome assessors, data analysts), and how	-	n/a
	17b	If blinded, circumstances under which unblinding is permissible, and procedure for revealing a participant's allocated intervention during the trial	-	n/a
**Methods: Data collection, management, and analysis**				
Data collection methods	18a	Plans for assessment and collection of outcome, baseline, and other trial data, including any related processes to promote data quality (eg, duplicate measurements, training of assessors) and a description of study instruments (eg, questionnaires, laboratory tests) along with their reliability and validity, if known. Reference to where data collection forms can be found, if not in the protocol	-	12, 26, 28, 30 - 34, 47
	18a.1		Describe what is known about the responsiveness of the study instruments in a population similar to the study sample	30
	18a.2		Describe who will assess the outcome (eg, nurse, parent)	33
	18b	Plans to promote participant retention and complete follow-up, including list of any outcome data to be collected for participants who discontinue or deviate from intervention protocols	-	22, 44

For peer review only - http://bmjopen.bmj.com/site/about/guidelines.xhtml

Section	Item No.	SPIRIT 2013 Item	SPIRIT-Outcomes 2022 item	Location Reported[b]
Data management	19	Plans for data entry, coding, security, and storage, including any related processes to promote data quality (eg, double data entry; range checks for data values). Reference to where details of data management procedures can be found, if not in the protocol	-	37, 39
Statistical methods	20a	Statistical methods for analysing primary and secondary outcomes. Reference to where other details of the statistical analysis plan can be found, if not in the protocol	-	45
	20a.1		Describe any planned methods to account for multiplicity in the analysis or interpretation of the primary and secondary outcomes (eg, coprimary outcomes, same outcome assessed at multiple time points, or subgroup analyses of an outcome)	n/a
	20b	Methods for any additional analyses (eg, subgroup and adjusted analyses)	-	n/a
	20c	Definition of analysis population relating to protocol non-adherence (eg, as randomised analysis), and any statistical methods to handle missing data (eg, multiple imputation)	-	45
**Methods: Monitoring**				
Data monitoring	21a	Composition of data monitoring committee (DMC); summary of its role and reporting structure; statement of whether it is independent from the sponsor and competing interests; and reference to where further details about its charter can be found, if not in the protocol. Alternatively, an explanation of why a DMC is not needed	-	9, 46
	21b	Description of any interim analyses and stopping guidelines, including who will have access to these interim results and make the final decision to terminate the trial	-	n/a
Harms	22	Plans for collecting, assessing, reporting, and managing solicited and spontaneously reported adverse events and other unintended effects of trial interventions or trial conduct	-	44 - 45

For peer review only - http://bmjopen.bmj.com/site/about/guidelines.xhtml

Section	Item No.	SPIRIT 2013 Item	SPIRIT-Outcomes 2022 item	Location Reported[b]
Auditing	23	Frequency and procedures for auditing trial conduct, if any, and whether the process will be independent from investigators and the sponsor	-	42
**Ethics and dissemination**				
Research ethics approval	24	Plans for seeking research ethics committee/institutional review board (REC/IRB) approval	-	47
Protocol amendments	25	Plans for communicating important protocol modifications (eg, changes to eligibility criteria, outcomes, analyses) to relevant parties (eg, investigators, REC/IRBs, trial participants, trial registries, journals, regulators)	-	2
Consent or assent	26a	Who will obtain informed consent or assent from potential trial participants or authorised surrogates, and how (see Item 32)	-	24 - 25
	26b	Additional consent provisions for collection and use of participant data and biological specimens in ancillary studies, if applicable	-	n/a
Confidentiality	27	How personal information about potential and enrolled participants will be collected, shared, and maintained in order to protect confidentiality before, during, and after the trial	-	40, 47 - 48
Declaration of interests	28	Financial and other competing interests for principal investigators for the overall trial and each study site	-	n/a
Access to data	29	Statement of who will have access to the final trial dataset, and disclosure of contractual agreements that limit such access for investigators	-	48 - 49
Ancillary and post-trial care	30	Provisions, if any, for ancillary and post-trial care, and for compensation to those who suffer harm from trial participation	-	n/a
Dissemination policy	31a	Plans for investigators and sponsor to communicate trial results to participants, healthcare professionals, the public, and other relevant groups (eg, via publication, reporting in results databases, or other data sharing arrangements), including any publication restrictions	-	50
	31b	Authorship eligibility guidelines and any intended use of professional writers	-	50

For peer review only - http://bmjopen.bmj.com/site/about/guidelines.xhtml

Section	Item No.	SPIRIT 2013 Item	SPIRIT-Outcomes 2022 item	Location Reported[b]
	31c	Plans, if any, for granting public access to the full protocol, participant-level dataset, and statistical code	-	-
**Appendices**				
Informed consent materials	32	Model consent form and other related documentation given to participants and authorised surrogates	-	-
Biological specimens	33	Plans for collection, laboratory evaluation, and storage of biological specimens for genetic or molecular analysis in the current trial and for future use in ancillary studies, if applicable	-	n/a

[a]It is strongly recommended that this checklist be read in conjunction with the SPIRIT (Standard Protocol Items: Recommendations for Interventional Trials) Statement paper for important clarification on the items. Amendments to the protocol should be tracked and dated. The SPIRIT checklist is copyrighted by the SPIRIT Group under the Creative Commons "Attribution-NonCommercial-NoDerivs 3.0 Unported" license and is reproduced with permission.
[b]Indicates page numbers and/or manuscript location: to be completed by authors.

For peer review only

For peer review only - http://bmjopen.bmj.com/site/about/guidelines.xhtml

BMJ Open

A pilot study to evaluate the use of remote patient monitoring to guide the timing of valve intervention in patients with severe asymptomatic aortic stenosis (APRAISE-AS): study protocol for a randomised controlled trial delivered in two tertiary cardiac centres in the United Kingdom

Journal:	*BMJ Open*
Manuscript ID	bmjopen-2024-086587.R1
Article Type:	Protocol
Date Submitted by the Author:	02-Apr-2024

Complete List of Authors:	Khan, Nazish; University Hospitals Birmingham NHS Foundation Trust, Department of Cardiology (QEHB)<br>Steeds, Richard; Queen Elizabeth Hospital, Department of Cardiology<br>Kyte, Derek; University of Worcester,<br>Fabritz, Larissa; University of Birmingham, Institute of Cardiovascular Sciences<br>Collis, Philip; University of Birmingham, Centre for Patient Reported Outcomes Research and Institute of Applied Health Research<br>Chua, Winnie; The University of Birmingham, Institute of Cardiovascular Sciences<br>Stubbs, Clive; University of Birmingham<br>Mehta, Samir; University of Birmingham, Birmingham Clinical Trials Unit<br>Sun, Yongzhong; University of Birmingham, Birmingham Clinical Trials Unit (BCTU)<br>Nulty, Mary; University of Birmingham, Birmingham Clinical Trials Unit<br>Kirkham, Katie; University of Birmingham, Birmingham Clinical Trials Unit<br>Cotton, James; The Royal Wolverhampton Hospitals, Department of Cardiology

**Primary Subject Heading**:	Cardiovascular medicine
Secondary Subject Heading:	Cardiovascular medicine
Keywords:	Patient Reported Outcome Measures, Valvular heart disease < CARDIOLOGY, Patient-Centered Care, Patient Satisfaction

SCHOLARONE™ Manuscripts

A pilot study to evaluate the use of remote patient monitoring to guide the timing of valve intervention in patients with severe asymptomatic aortic stenosis (APRAISE-AS): study protocol for a randomised controlled trial delivered in two tertiary cardiac centres in the United Kingdom

Contributors: Nazish Khan[1,2], Richard P. Steeds[1,2], Derek Kyte[3,4], Larissa Fabritz[1,5], Phil Collis[2], Winnie Chua[1], Clive Stubbs[6] Samir Mehta[6], Yongzhong Sun[6], Mary Nulty[6], Katie Kirkham[6], James M. Cotton[7,8]

1. Department of Cardiology (QEHB), University Hospitals Birmingham NHS Foundation Trust, UK
2. Institute of Cardiovascular Sciences, University of Birmingham, Birmingham, UK
3. School of Allied Health and Community, University of Worcester, Worcester, UK
4. Centre for Patient Reported Outcomes Research, Institute for Applied Health Research, University of Birmingham, Birmingham, UK
5. University Center of Cardiovascular Sciences & Department of Cardiology, University Heart and Vascular Center, University Hospital Hamburg Eppendorf, Hamburg, Germany
6. Birmingham Clinical Trials Unit, Institute for Applied Health Research, University of Birmingham, Birmingham, UK
7. Department of Cardiology, The Royal Wolverhampton Hospitals NHS Trust

Corresponding author: Nazish Khan E-mail: nazish.khan@nhs.net

Word count (including abstract, excluding references: 4,334)

Abstract

Introduction: Aortic stenosis (AS) is common affecting >13% of adults over the age of 75 years. In people who develop symptoms, without valve replacement, prognosis is dismal with mortality as high as 50% at 1 year. In asymptomatic patients, the timing of valve intervention is less well defined and a strategy of watchful waiting is recommended. Many, however, may develop symptoms and attribute this to age related decline, rather than worsening AS. Timely intervention in asymptomatic severe AS is critical, since delayed intervention often results in poor outcomes. Proactive surveillance of symptoms, quality of life and functional capacity should enable timely identification of people who will benefit from aortic valve replacement. There are no data however, to support the clinical and cost effectiveness of such an approach in a healthcare setting in the United Kingdom. The aim of this pilot trial is to test the feasibility of a full-scale randomised controlled trial to determine the utility of proactive surveillance in people with asymptomatic severe AS to guide the timing of intervention.

For peer review only

Methods and analysis: APRAISE-AS is a multi-centre, non-blinded, two-arm, parallel group randomised controlled trial of up to 66 participants aged >18 years with asymptomatic severe AS. Participants will be randomised to either standard care or standard care supplemented with the APRAISE-AS intervention. Primary outcomes will capture; adherence to and participant acceptability of the intervention, recruitment and retention rates, and completeness of data collection. The findings will be used to inform the sample size and most appropriate outcome measure(s) for a full-scale RCT and health economic evaluation.

Ethics and dissemination: Ethical approval was granted by the Black Country REC, reference: 22/WM/0214. Results will be submitted for publication in peer reviewed journals and disseminated at local, regional and national meetings where appropriate.

Trial registration number(s): ISRCTN reference: 19413194 registered 14.07.2023.

Strengths and limitations of this study:

- The study uses a randomised controlled trial design to assess the feasibility of how subjective (symptoms & health related quality of life) and objective (functional capacity) patient reported data might supplement standard care to inform the timeliness of aortic valve replacement.
- The intervention has been developed via a process of participatory design in collaboration with patients and clinicians.
- The intervention provides an innovative way of using digital technology in patients with heart valve disease (aortic stenosis), to support patient engagement and improve the patient pathway through facilitating enhanced remote patient monitoring, near real time clinician assessment and risk stratification to inform the optimum time at which valve intervention should take place.
- The study is not powered to determine clinical outcomes but is designed to determine the feasibility of conducting a full-scale RCT to understand the clinical utility and cost-effectiveness of integrating an innovative approach to proactive, remote patient monitoring into the clinical management pathway of people with asymptomatic severe AS.

Key words: asymptomatic severe aortic stenosis, valve replacement, symptoms, patient reported outcome measures, functional capacity

INTRODUCTION

For peer review only

Heart valve disease (HVD) affects 1.5 million people over the age of 65 years in the United Kingdom and is an important cause of morbidity and mortality (1). Aortic stenosis (AS) is the most common acquired form of HVD encountered in clinical practice (2). The overall burden of disease in the general population is substantial with an incidence as high as 13% in those aged ≥ 75 years (3). Prevalence and associated healthcare costs secondary to AS are likely to rise in view of an increasingly ageing, frail and multi-morbid population.

AS is characterised by insidious thickening and calcification of a native aortic valve and is an active process driven by lipid accumulation, inflammation and fibrosis (4, 5). Whilst the underlying pathophysiology of the condition is similar to the process of atherosclerosis, there are no proven medical treatments that can limit disease progression and AS is treated by aortic valve replacement (AVR) (2).

Severity of AS is determined by echocardiographic parameters including the maximal velocity, mean pressure gradient and aortic valve area (6). Intervention is mandated in severe AS following the development of symptoms including breathlessness, dizziness, blackouts, chest pain and palpitations. Prognosis is dismal following the development of symptoms, with 1-year mortality as high as 50%, and prompt intervention is therefore critical (7) (6).

In asymptomatic patients however, the timing of valve intervention remains less well defined. A strategy of watchful waiting is adopted, and patients are counselled on the importance of immediate reporting should symptoms develop. Regular clinical surveillance is mandatory and a full physical assessment and echocardiogram should be undertaken at least every six months (6). The slowly progressive nature of AS combined with the advanced age of the affected population, however, predispose to underreporting and/or underestimation of symptoms (8). Risks associated with a watchful waiting approach include irreversible myocardial damage, sudden cardiac death (with an incidence of 1.5% per year), delayed symptom reporting and dying whilst awaiting valvular intervention (9, 10).

Unmet need

The optimal timing of valve intervention in asymptomatic severe AS is currently under investigation in a number of randomised controlled clinical trials; the outcomes of EARLY-TAVR and EVOLVED, will provide insight into whether proactive surveillance, diagnostic imaging and an understanding of the

For peer review only

optimum window of opportunity can be implemented in clinical practice to guide the timing of intervention in patients with asymptomatic severe AS (11, 12).

Objective

To date there have been no high-quality, multicentre RCTs to test the clinical and cost-effectiveness of whether proactive symptom surveillance and remote patient monitoring using a software application ("app") in addition to a traditional "watchful waiting" strategy in adult patients with asymptomatic severe AS allows for earlier identification of symptoms and timelier aortic valve intervention leading to improved clinical outcomes. However, before a large RCT can be undertaken, a pilot trial is required to inform the most appropriate outcome measures and sample size for a full-scale RCT. Therefore the APRAISE-AS study will pilot the trial protocol, in order to assess the feasibility and potential refinement of the trial design and/or trial intervention prior to undertaking a larger randomised controlled trial investigating whether proactive reporting of symptoms, definitive disease specific patient reported outcome measures (PROMs) and assessment of frailty in addition to standard care can be used to help inform the timeliness of valve intervention in patients with asymptomatic severe aortic stenosis.

METHODS

Trial design and setting

This protocol adheres to guidance from the Standard Protocol Items: Recommendations for Interventional Trials (SPIRIT) and a SPIRIT checklist is included in an accompanying supplementary file (13, 14). The APRAISE-AS pilot trial is a multi-centre, non-blinded, two-arm, parallel group randomised controlled pilot trial of up to 66 participants aged 18 years or over with asymptomatic severe AS. The pilot trial will be conducted in two tertiary cardiology and cardiothoracic surgery Heart Valve centres in the West Midlands. Participants will be randomised in a 1:1 ratio to receive either standard care or standard care supplemented with the APRAISE-AS intervention. The study trial schema is described in figure 1.

Recruitment and eligibility

Recruitment commenced in February 2023 in The Royal Wolverhampton NHS Trust and October 2023 at the Queen Elizabeth Hospital Birmingham and closed in January 2024 and February 2024 respectively. Patients were invited to participate if they had a diagnosis of asymptomatic severe AS and if they met the following eligibility criteria:

For peer review only

Inclusion criteria

- Patients diagnosed with asymptomatic severe AS of a native valve, in line with current international guideline and defined as either:
    - **(a)** Aortic valve area <1cm^2 and mean pressure gradient >40mmHg and LVEF >50% (in the last 6 months), OR
    - **(b)** Aortic valve area <1cm^2 and mean pressure gradient ≤ 40mmHg, stroke volume index ≤ 35ml/m^2 and LVEF >50% (in the last 6 months)
- Able to give written informed consent
- Age ≥ 18 years
- Able to access and use app-based programmes

Exclusion criteria

- Lack of capacity to understand the nature of the study and possible consequences as determined by their attending clinician
- Moderate or more severe disease of other valves e.g. mitral stenosis/regurgitation
- Co-morbidities that in the opinion of the cardiac surgeon/cardiologist preclude successful valve intervention e.g. life expectancy of less than 2 years

Participation identification, recruitment and informed consent

Adult patients with a confirmed diagnosis of severe asymptomatic AS of a native valve were recruited. Patients were identified for eligibility and approached for recruitment via one of three routes: (i) following automated e-mail alert for people who meet echocardiographic parameters associated with severe AS, (ii) new referrals seen in out-patient clinics or following identification at ward level, or (iii) from cardiac investigation department echocardiogram diagnostic lists. Existing patients known to the respective cardiac research teams were also be approached for recruitment. Following confirmation of eligibility by the cardiovascular research team (clinicians/pharmacists/nurses), potential participants were contacted by telephone to gauge interest in the trial and request permission for a participant information sheet to be sent to them either via e-mail or by post. In addition, potential participants were also be given a participant information sheet during their clinic appointment. The research team gave potential participants sufficient time to review the information provided, discuss the trial with family members and/or their GP. The Chief Investigator/site Principal Investigators ensured that participants fully understand the aim of the trial, the intervention and potential benefits and drawbacks associated with participation. On receiving an expression of interest

For peer review only

from the potential participant, they were invited to an outpatient appointment where informed consent for participation was obtained in addition to completion of baseline assessments of symptom status, functional capacity and quality of life were undertaken prior to randomisation. Participants were reminded that participation is voluntary and they may withdraw from the trial at any time and it will not affect the care they receive.

Randomisation

Following baseline assessments, participants were randomised at the level of the individual in a 1:1 ratio to either standard care (control arm) or standard care supplemented with the APRAISE-AS intervention (intervention arm). Randomisation was undertaken by Birmingham Clinical Trials Unit (BCTU) using a computer-generated sequence created by a programmer independent to the study team. A minimisation algorithm has been used within the randomisation system to ensure balance in the intervention allocations over the following variables:

- Age ('< 50 years' and '≥ 50 years')
- Sex ('male' and 'female')
- Ethnicity ('white' and 'non-white')

To avoid the possibility of the intervention allocation becoming predictable, a random element will be included in the algorithm (unspecified here), so that each participant has a chance of being randomised to the opposite intervention that they would have otherwise received.

Interventions

Participants assigned to standard care (control arm) will continue to receive usual care which includes a 6 monthly valve clinical review and surveillance echocardiogram (as part of a watchful waiting strategy). For participants assigned to the intervention arm, standard care will be supplemented with the APRAISE-AS intervention. The APRAISE-AS intervention includes an app and a clinician's portal which has been developed via a process of participatory design in collaboration with our patients and clinicians, which will collect both subjective and objective data on a patients' health and well-being following their diagnosis of asymptomatic severe AS. This information will be presented to the healthcare team via a bespoke platform which allows near real-time surveillance of the patients' healthcare data (Clinician's Portal). The aim of the app is to facilitate clinical prioritisation of patients who demonstrate symptoms or early signs of decompensation to allow for escalation of treatment and/or earlier intervention. These self-reported patient tests will be used to supplement routine clinical appointments.

For peer review only

For participants randomised to standard care plus the APRAISE-AS intervention, a member of the research team will install and provide access to the app on the participant's own, or a Trust sourced, device. Over a 12-month period, participants will use the app to self-report information relating to:

- Symptoms [(assessed using New York Heart Association and Canadian Cardiovascular System classification) dyspnoea/fatigue/palpitations/angina and syncope] on a weekly basis.
- Frailty/functional capacity (Timed Up and Go test) every two weeks [frailty, assessed using gait speed, is known to correlate with major morbidity and mortality after valve surgery and provides a continuous reproducible measure allowing for an assessment of functional status over time (15-17)].
- Disease specific PROMs using the Kansas City Cardiomyopathy Questionnaire (KCCQ) once a month [KCCQ has been studied extensively in the setting of AS and has been found to be a reliable, reproducible and valid measure of symptoms, functional status, quality of life and prognosis (18)].

Participants will receive automated reminders prior to submitting a self-assessment via the app and 24 hours after a failure to submit their report, if necessary. Severe symptoms noted by the patient will trigger an email alert to the APRAISE-AS clinical team (dedicated monitored email inbox) and a simultaneous patient notification advising them to make contact with their clinical team during office hours (or to use standard NHS support mechanisms outside of these hours). The clinical team will monitor for notifications and respond in line with standard care recommendations during office hours. Actions taken in response to an alert will be logged in the patient's electronic healthcare record by a member of the APRAISE-AS clinical team (including members of the cardiovascular research team).

Outcome measures and study procedures

The primary aim of this study is to pilot the trial protocol and assess the feasibility and potential refinement of the trial design prior to undertaking a larger randomised controlled trial (RCT) investigating whether proactive surveillance/reporting of symptoms, definitive disease specific PROMs and assessment of frailty in addition to standard care can be used to help inform the timeliness of valve intervention in patients with asymptomatic severe AS. In addition, the pilot will allow for refinement of the trial design and/or trial intervention prior to undertaking a larger multi-centre RCT which will assess the clinical and cost-effectiveness of remote patient monitoring in this setting. The aim of using the outcomes to enable pathway transformation and service redesign through the incorporation of remote monitoring into the clinical management pathway for people with asymptomatic severe AS.

For peer review only

The pilot trial will:

- Test and pilot the trial protocol (including recruitment and retention rates, data collection processes, data completeness and adherence to the APRAISE-AS intervention);
- Inform selection of the most appropriate primary outcome measure for a full-scale multicentre RCT;
- Provide data to help estimate the sample size for a full-scale multicentre RCT;
- Provide a platform to develop and pilot the processes to capture costs and outcomes to inform the health economic evaluation for a full-scale multicentre RCT;
- Determine key participation criteria for centre involvement in a full-scale RCT.

Primary outcomes

In addition to piloting the trial protocol, the pilot study will assess:

- The utility of the "app" using quantitative (e.g. usage data, data completeness) and qualitative (interviews) data
- Engagement with and acceptability of the intervention
- Recruitment rate (per month)
- Number of withdrawals and losses to follow-up (with reasons)

Secondary outcomes

Data will be collated on outcomes measures that we plan to collect in the definitive RCT (e.g. primary outcome in the phase III trial at 12 months follow up). Whilst the pilot trial is not powered to detect meaningful differences in these outcomes, collection of these data will provide insight into how readily they can be collected in the context of a larger multicentre RCT. Data will be collected on:

- Disease-specific PROMs assessed using KCCQ
- Frailty/functional capacity measured using the Timed Up and Go test
- Time to heart valve replacement
- In-hospital mortality in those who undergo valve replacement
- Mortality at 30 days post-randomisation
- Mortality at 1 year post-randomisation
- Incidence of stroke in those who undergo valve replacement
- Incidence of myocardial infarction in those who undergo valve replacement
- Left ventricular ejection fraction at 6 and 12 months
- Incidence of unplanned hospital admissions

For peer review only

All study staff and participants will be invited to complete a trial process questionnaire at the end of the study, which will evaluate aspects surrounding:

- The extent and frequency of data collection
- Randomisation procedure
- Acceptability of the intervention (e.g. for patients using the mobile app rating score (MARS) tool.
- Appropriateness of the frequency of APRAISE-AS reporting, alert thresholds and subsequent management.
- The utility of the clinician's portal and embedding remote monitoring into the clinical management pathway.

Study Procedures

During the baseline visit participants' demographic (age, gender and ethnicity) and biomarker (creatinine, creatinine clearance) data will be collected. Baseline assessment of symptoms, functional capacity (using the Timed Up and Go test) and health related quality of life (using the disease specific KCCQ) will be undertaken. Undertaking an objective assessment of frailty using a parameter such as gait speed, which correlates with major morbidity and mortality after valve surgery, provides a continuous reproducible measure allowing for an assessment of functional status over time (15-17). In addition, a comprehensive determination of health-related quality of life for patients undergoing valvular intervention, incorporating heart failure specific measures is recommended (15). The disease specific patient reported outcome measure (PROM) Kansas City Cardiomyopathy Questionnaire (KCCQ) has been studied extensively in the setting of AS and has been found to be a reliable, reproducible and valid measure of symptoms, functional status, quality of life and prognosis (18).

As described in table 1, all participants will complete the timed up and go test and paper versions of the KCCQ and symptom tracker questionnaires (in clinic) at baseline (prior to randomisation), and when attending the two subsequent follow up visits. For participants assigned to the standard care arm, post-randomisation timed up and go, symptom and health status questionnaires will only be completed at the routine 6- and 12-months follow-up appointments. For those participants assigned to the intervention arm, this information will also be collected via the app at pre-specified frequencies post-randomisation as well as during their routine 6- and 12-month follow-up appointments.

For peer review only

Adherence with the APRAISE-AS intervention will be assessed by calculating the number and percentage (with 95% CI) of patients who complete the symptom surveillance, frailty and quality of life assessments as scheduled. To be considered adherent, patients will need to have submitted their report within 24 hours of the scheduled time-point. Incomplete submissions (i.e. with some questions not answered) will be accepted for the purpose of measuring adherence. Ad-hoc reports (i.e. those completed outside the scheduled reporting periods) will not contribute to the assessment of adherence, although we will assess the number of APRAISE-AS reports completed by each patient.

Table 1. Schedule of Assessments for KCCQ (PROMs), symptoms and Timed Up & Go (frailty) data capture

Assessment	Baseline/prior to randomisation	Once a week ± 2 days	Once every 2 weeks ± 2 days	Once a month ± 2 days	Month 6 ± 2 days	Month 12 ± 2 days	Ad-hoc reporting	Clinical site continuous daily monitoring (intervention arm)	Responding to severe symptoms* Clinical site continuous daily monitoring
Control Group									
Symptoms	x				x	x			x
Timed Up & Go	x				x	x			x
KCCQ	x				x	x			x
Intervention Group									
Symptoms	x	x			x	x	x	x	x
Timed Up & Go	x		x		x	x	x	x	x
KCCQ	x			x	x	x	x	x	x

*following submission of a severe symptom, the clinical team will document the action taken and outcome within the notes section in the clinician's portal and within the patient record used as per standard local clinical practice.

Sample size and justification

Since this is a pilot study a formal sample size calculation has not been performed. In order to obtain estimates of the parameters needed for sample size determination, in line with published recommendations for pilot studies, 30 patients are required (19, 20). Assuming a loss to follow-up rate of 10% over the 12-month follow up period, the study will require 33 patients to be randomised to each group, with a total of 66 patients overall. This will also allow for estimation of recruitment and retention rates with 95% CI maximum widths of 20% and 25%, respectively.

Analysis of outcome measures

For peer review only

A separate Statistical Analysis Plan (SAP) will be produced and will provide a more comprehensive description of the planned statistical analyses. A brief outline of the planned analyses is given below. The primary comparison groups will be composed of those randomised to standard care supplemented with the APRAISE-AS intervention (intervention group) versus those randomised to standard care (control group). All analyses will be based on the intention to treat (ITT) principle, i.e. all patients will be analysed in the treatment group to which they were randomised irrespective of compliance with the allocated treatment or other protocol deviation. The data analysis for this pilot trial will mainly be descriptive and focus on confidence interval estimation, with no hypothesis testing performed. The reporting and presentation of this trial will be in accordance with the CONSORT guidelines for randomised trials.

The pilot data will also help inform the selection of the most appropriate primary outcome measure for the main RCT and provide contributory data to facilitate estimation of the sample size required for the main RCT. Analysis methods will be chosen according to the data type of the outcome under investigation, in brief:

- *Continuous outcomes (e.g. quality of life)*: These data will be summarised using means and standard deviations, with differences in means with 95% confidence intervals reported. Longitudinal plots of the data over time will also be constructed for visual presentation of the data.
- *Categorical (dichotomous) outcomes (e.g. hospitalisation rates)*: The number of patients and percentages experiencing the event will be summarised between groups.
- *Time to Event outcomes (e.g. time to symptom development, valve replacement, mortality):* The numbers of patients and percentages experiencing the event will be summarised over time between groups. Kaplan-Meier survival curves will be constructed for visual presentation of time-to-event data.

Missing data and sensitivity analyses

Every attempt will be made to collect full follow-up data on all study patients; it is thus anticipated that missing data will be minimal. The assessment of missing data is an outcome measure of this pilot trial and forms part of the assessment of the feasibility of a future trial. The amount of missing data at outcome measure level will also be considered when assessing and identifying a suitable primary outcome for the future trial. As this is a pilot trial, no formal sensitivity analysis will be conducted.

Planned interim analysis

For peer review only

Since this is a pilot study a planned interim analysis will not be undertaken. However, the trial oversight committee will access to recruitment, retention and data collection information.

Planned final analysis

The primary analysis for the study will occur once all patients have completed their 12-month assessment and corresponding outcome data have been entered onto the study database (REDCap) and validated as being ready for analysis. This analysis will include data items up to and including the 12-month assessment and no further.

Health Economics

A health economic evaluation is not planned for this pilot trial. However, data will be collected to capture costs and outcomes to inform the health economic evaluation for a multi-centre full-scale RCT. Data will be collated in relation to cardiology clinical staff time and activity in response to APRAISE-AS notifications, including ad-hoc telephone consultations, in-patient's hospitalisations and onward referrals for consideration of aortic valve replacement, in addition to NHS costs attributable to maintenance of the APRAISE-AS clinician's portal. Where possible data on healthcare resource utilisation will be collected from both electronic patient records and case report forms either during study follow up visits or following response to a severe symptom alert.

Monitoring, trial management and oversight

The Chief Investigator has overall responsibility for the study. The Trial Management Group (TMG) consisting of the cardiovascular research team (including chief investigator and site principal investigators) will oversee day to day running of trial. Due to the small sample size and pilot nature of this trial, the Trial Steering and Data Monitoring Committees have been combined into a single Trial Oversight Committee (TOC). The TOC will provide overall oversight of the trial, including the practical aspects of the study, as well as ensuring that the study is run in a way which is both safe for the patients and provides appropriate feasibility data to the sponsor and investigators. The TOC will oversee monitoring of the trial data and make recommendations to the TMG on whether there are any ethical or safety reasons as to why the trial should not continue or whether it needs to be modified. To this end, data on safety will be supplied to the TOC during the trial and all reports will be supplied in confidence.

Data management

Electronic case report forms, with programmed range checks, will be entered online via a secure REDCap database administered by BCTU. Authorised staff require an individual secure login username

For peer review only

and password to access online data entry. All missing and ambiguous data are queried through the online system. The security of the System is governed by the policies of the University of Birmingham (Data Protection Registration number Z6195856), and each study site has arrangements in place for secure storage and processing of study data, in compliance with the University of Birmingham policies. The University carries appropriate Data Protection Registration coverage.

Audit

BCTU staff will monitor electronic case report forms (CRF) returns for data completion and will work closely with site delivery teams to ensure that informed consent forms (ICFs) and CRFs are returned in a timely manner. The BCTU team will also cross reference ICF and CRF content against the trial protocol to ensure compliance with protocol standards and consistency in and completeness of data reporting.

ETHICS AND DISSEMMINATION

The trial will be conducted in accordance with the UK Policy Framework for Health and Social Care Research and applicable UK Acts of Parliament and Statutory Instruments (and relevant subsequent amendments), which include the Data Protection Act 2018 and the Principles of GCP as set out in the UK Statutory Instrument (2004/1031; and subsequent amendments). The protocol has been submitted to and approved by West Midlands Black Country Research Ethics Committee (reference number 22/WM/0214) and the Health Research Authority on 05/12/2022. Each trial site will confirm local NHS Trust Research & Development department capacity and capability prior to commencing recruitment. All participants will provide written informed consent prior to participation in the trial. University of Birmingham are the sponsor for the trial.

For peer review only

The findings from the pilot trial will be used to inform the design of a full-scale multicentre RCT. Outputs from the trial will be submitted for publication in peer reviewed journals, presented at scientific conferences and disseminated to the public via patient charities/patient advocacy groups. The manuscript will be prepared by the Chief Investigator and authorship will be determined by the trial publication policy. Any secondary publications and presentations prepared by Investigators must be reviewed and approved by the TMG. Manuscripts must be submitted to the TMG in a timely fashion and in advance of being submitted for publication, to allow time for review and resolution of any outstanding issues. Authors must acknowledge that the trial was performed with the support of the sponsor (University of Birmingham) and BCTU. Intellectual property rights will be addressed in the Clinical Study Site Agreement between Sponsor and site.

Patient and Public Involvement

Members of a local hospital patient charity which comprised people living with heart disease were initially involved providing constructive feedback regarding the research question and study design. A patient advisory group (PAG) was established to inform the development, testing and refinement of the APRAISE-AS app via a process of participatory design. The PAG supported user testing of the app and their input guided the frequency of reporting for the symptoms, timed up and go and health related quality of life assessments to ensure acceptability in terms of burden from a patient's perspective. The PAG also reviewed all patient facing materials and a member of the PAG also sits on the TMG. Our PPIE co-applicant will support dissemination of trial findings among relevant patient groups.

Data Availability Statement

Data are available upon reasonable request. Data requesters should e-mail the corresponding author for the paper to request the relevant data.

Author contributions:

NK is the Chief Investigator and takes final responsibility for the study design, conduct and decision to submit for publication. NK prepared the first draft of the protocol with approval from all authors. JMC is co-Chief Investigator and site Principal Investigator, RS is a site Principal Investigator, both provided clinical expertise. CS, MN, KK provided trials expertise. SM & YS (statisticians) designed and wrote the statistical analysis plan. DK, LF, WC provided expertise in terms of PROMs trial methodology and the delivery of remote monitoring in the context of a clinical trial. PC provided expertise in terms of PROMs and PPIE. All investigators provided advice and critical input on the study design. All authors critically revised the manuscript for its important intellectual content. All authors read and approved the final manuscript.

Funding statement

This study is funded by the National Institute for Health and Care Research (NIHR) under its Research for Patient Benefit (RfPB) Programme (Grant Reference Number NIHR202810) and sponsored by the University of Birmingham (reference: RG_22-094).

Disclaimer

For peer review only

The views expressed are those of the author(s) and not necessarily those of the NIHR or the Department of Health and Social Care.

NK and JC were also financially supported by the West Midlands Clinical Research Network and Wolverhampton Coronary Care After Support Patient Group. The views expressed are those of the author(s) and not necessarily those of the NIHR or the Department of Health and Social Care.

Competing interests' statement:

LF reports grants from the BHF (AA/18/2/34218), EU Horizon 2020 CATCH ME (grant agreement number 633196), MAESTRIA (grant agreement number 965286), AFFECT-EU (grant agreement number 847770, NIHR award 1002898 (APRAISE-AS) at University of Birmingham, and German Center for Cardiovascular Research (DZHK). LF has also received institutional research grants from governmental funding agencies, charities and companies in biomedical research. LF is part of the scientific advisory board of a patient initiative.

DK reports grants from the NIHR and Kidney Research UK outside the submitted work. NK reports grants from the NIHR.

Patient consent

Not required

References:

1. Voice HV. Heart Valve Voice https://heartvalvevoice.com2019 [Available from: https://heartvalvevoice.com/heart-valve-disease/prevalence.
2. Osnabrugge RL, Mylotte D, Head SJ, Van Mieghem NM, Nkomo VT, LeReun CM, et al. Aortic stenosis in the elderly: disease prevalence and number of candidates for transcatheter aortic valve replacement: a meta-analysis and modeling study. Journal of the American College of Cardiology. 2013;62(11):1002-12.
3. Kwok CS, Bagur R, Rashid M, Lavi R, Cibelli M, de Belder MA, et al. Aortic stenosis and non-cardiac surgery: A systematic review and meta-analysis. International journal of cardiology. 2017;240:145-53.
4. Otto CM. Valvular aortic stenosis: disease severity and timing of intervention. Journal of the American College of Cardiology. 2006;47(11):2141-51.
5. Everett RJ, Clavel MA, Pibarot P, Dweck MR. Timing of intervention in aortic stenosis: a review of current and future strategies. Heart. 2018;104(24):2067-76.
6. Vahanian A, Beyersdorf F, Praz F, Milojevic M, Baldus S, Bauersachs J, et al. 2021 ESC/EACTS Guidelines for the management of valvular heart disease. Eur J Cardiothorac Surg. 2021;60(4):727-800.
7. Leon MB, Smith CR, Mack M, Miller DC, Moses JW, Svensson LG, et al. Transcatheter aortic-valve implantation for aortic stenosis in patients who cannot undergo surgery. N Engl J Med. 2010;363(17):1597-607.

For peer review only

8. Tribouilloy C, Rusinaru D, Bohbot Y, Marechaux S, Vanoverschelde JL, Enriquez-Sarano M. How Should Very Severe Aortic Stenosis Be Defined in Asymptomatic Individuals? J Am Heart Assoc. 2019;8(3):e011724.
9. Rosenhek R, Zilberszac R, Schemper M, Czerny M, Mundigler G, Graf S, et al. Natural history of very severe aortic stenosis. Circulation. 2010;121(1):151-6.
10. Taniguchi T, Morimoto T, Shiomi H, Ando K, Kanamori N, Murata K, et al. Initial Surgical Versus Conservative Strategies in Patients With Asymptomatic Severe Aortic Stenosis. Journal of the American College of Cardiology. 2015;66(25):2827-38.
11. Généreux P. Evaluation of Transcatheter Aortic Valve Replacement Compared to SurveilLance for Patients With AsYmptomatic Severe Aortic Stenosis (EARLY TAVR) ClinicalTrials.gov: ClinicalTrials.gov; 2019 [Available from: https://clinicaltrials.gov/ct2/show/NCT03042104.
12. Dweck M. Early Valve Replacement Guided by Biomarkers of LV Decompensation in Asymptomatic Patients With Severe AS (EVoLVeD) ClinicalTrials.gov2019 [Available from: https://clinicaltrials.gov/ct2/show/NCT03094143.
13. Chan AW, Tetzlaff JM, Gotzsche PC, Altman DG, Mann H, Berlin JA, et al. SPIRIT 2013 explanation and elaboration: guidance for protocols of clinical trials. BMJ. 2013;346:e7586.
14. Chan AW, Tetzlaff JM, Altman DG, Laupacis A, Gotzsche PC, Krleza-Jeric K, et al. SPIRIT 2013 statement: defining standard protocol items for clinical trials. Ann Intern Med. 2013;158(3):200-7.
15. Kappetein AP, Head SJ, Genereux P, Piazza N, van Mieghem NM, Blackstone EH, et al. Updated standardized endpoint definitions for transcatheter aortic valve implantation: the Valve Academic Research Consortium-2 consensus document. European heart journal. 2012;33(19):2403-18.
16. Afilalo J, Lauck S, Kim DH, Lefevre T, Piazza N, Lachapelle K, et al. Frailty in Older Adults Undergoing Aortic Valve Replacement: The FRAILTY-AVR Study. Journal of the American College of Cardiology. 2017;70(6):689-700.
17. Sepehri A, Beggs T, Hassan A, Rigatto C, Shaw-Daigle C, Tangri N, et al. The impact of frailty on outcomes after cardiac surgery: a systematic review. J Thorac Cardiovasc Surg. 2014;148(6):3110-7.
18. Arnold SV, O'Brien SM, Vemulapalli S, Cohen DJ, Stebbins A, Brennan JM, et al. Inclusion of Functional Status Measures in the Risk Adjustment of 30-Day Mortality After Transcatheter Aortic Valve Replacement: A Report From the Society of Thoracic Surgeons/American College of Cardiology TVT Registry. JACC Cardiovasc Interv. 2018;11(6):581-9.
19. Lancaster GA, Dodd S, Williamson PR. Design and analysis of pilot studies: recommendations for good practice. J Eval Clin Pract. 2004;10(2):307-12.
20. Browne RH. On the use of a pilot sample for sample size determination. Stat Med. 1995;14(17):1933-40.

For peer review only

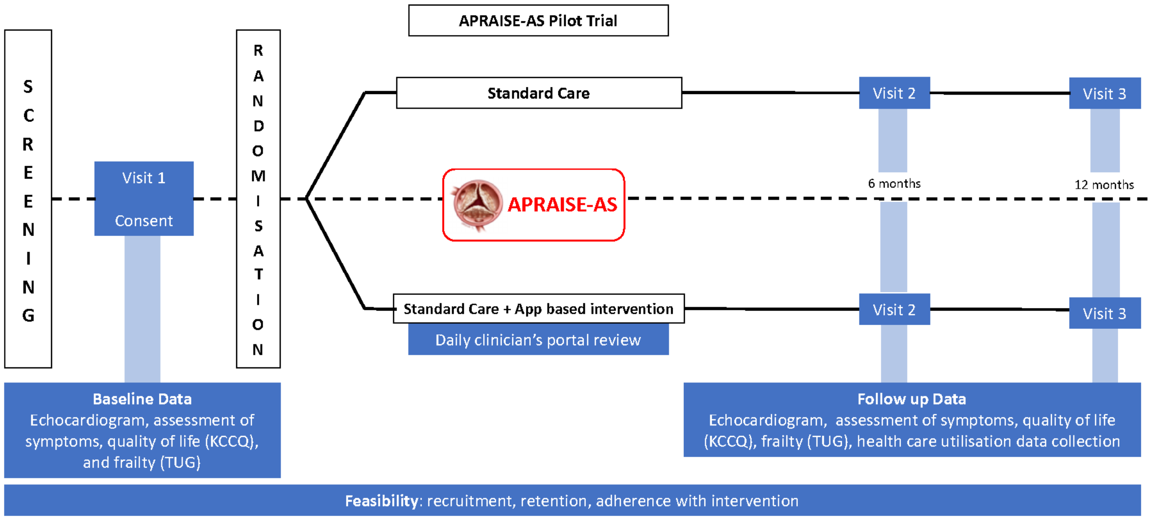

Inclusion Criteria

- Patients diagnosed with asymptomatic sAS of a native valve, with either:

(a) Aortic valve area <1cm2 and mean pressure gradient >40mmHg and LVEF >50% (in the last 6 months) OR
(b) Severe AS (low flow, low gradient): Aortic valve area <1cm2 and mean pressure gradient ≤ 40mmHg and LVEF >50% and SVi ≤ 35mL/m2 (in the last 6 months)

- Able to give written informed consent
- Age ≥18 years
- Able to access and use app-based programmes (using the English language)

Exclusion Criteria

- Lack of capacity to understand the nature of the study and possible consequences
- Significant disease of other valves e.g. mitral stenosis/regurgitation
- Co-morbidities that in the opinion of the cardiac surgeon/cardiologist preclude successful valve intervention e.g. life expectancy of less than 2 years

SPIRIT | CONSORT OUTCOMES Reporting Guidelines for Trial Outcomes

SPIRIT-Outcomes 2022 Checklist (for combined completion of SPIRIT 2013 and SPIRIT-Outcomes 2022 items)[a]

Section	Item No.	SPIRIT 2013 Item	SPIRIT-Outcomes 2022 item	Location Reported[b]
**Administrative information**				
Title	1	Descriptive title identifying the study design, population, interventions, and, if applicable, trial acronym	-	1
Trial registration	2a	Trial identifier and registry name. If not yet registered, name of intended registry	-	1
	2b	All items from the World Health Organization Trial Registration Data Set	-	n/a
Protocol version	3	Date and version identifier	-	1
Funding	4	Sources and types of financial, material, and other support	-	2
Roles and responsibilities	5a	Names, affiliations, and roles of protocol contributors	-	5 -8
	5b	Name and contact information for the trial sponsor	-	5
	5c	Role of study sponsor and funders, if any, in study design; collection, management, analysis, and interpretation of data; writing of the report; and the decision to submit the report for publication, including whether they will have ultimate authority over any of these activities	-	2, 46
	5d	Composition, roles, and responsibilities of the coordinating centre, steering committee, endpoint adjudication committee, data management team, and other individuals or groups overseeing the trial, if applicable (see Item 21a for data monitoring committee)	-	3,4, 8,9, 46
**Introduction**				
Background and rationale	6a	Description of research question and justification for undertaking the trial, including summary of relevant studies (published and unpublished) examining benefits and harms for each intervention	-	12, 20-22
	6b	Explanation for choice of comparators	-	22-23
Objectives	7	Specific objectives or hypotheses	-	12, 22-23

For peer review only - http://bmjopen.bmj.com/site/about/guidelines.xhtml

Section	Item No.	SPIRIT 2013 Item	SPIRIT-Outcomes 2022 item	Location Reported[b]
Trial design	8	Description of trial design including type of trial (eg, parallel group, crossover, factorial, single group), allocation ratio, and framework (eg, superiority, equivalence, noninferiority, exploratory)	-	12 - 13, 14, 23 - 24
**Methods: Participants, interventions, and outcomes**				
Study setting	9	Description of study settings (eg, community clinic, academic hospital) and list of countries where data will be collected. Reference to where list of study sites can be obtained	-	12,
Eligibility criteria	10	Inclusion and exclusion criteria for participants. If applicable, eligibility criteria for study centres and individuals who will perform the interventions (eg, surgeons, psychotherapists)	-	24
Interventions	11a	Interventions for each group with sufficient detail to allow replication, including how and when they will be administered (for specific guidance see TIDieR checklist and guide)	-	12 - 13, 28
	11b	Criteria for discontinuing or modifying allocated interventions for a given trial participant (eg, drug dose change in response to harms, participant request, or improving/worsening disease)	-	29
	11c	Strategies to improve adherence to intervention protocols, and any procedures for monitoring adherence (eg, drug tablet return, laboratory tests)	-	n/a
	11d	Relevant concomitant care and interventions that are permitted or prohibited during the trial	-	24
Outcomes	12	Primary, secondary, and other outcomes, including the specific measurement variable (eg, systolic blood pressure), analysis metric (eg, change from baseline, final value, time to event), method of aggregation (eg, median, proportion), and time point for each outcome. Explanation of the clinical relevance of chosen efficacy and harm outcomes is strongly recommended	-	12 - 13, 29 - 30, 20

For peer review only - http://bmjopen.bmj.com/site/about/guidelines.xhtml

Section	Item No.	SPIRIT 2013 Item	SPIRIT-Outcomes 2022 item	Location Reported[b]
	12.1		Provide a rationale for the selection of the domain for the trial's primary outcome	22
	12.2		If the analysis metric for the primary outcome represents within-participant change, define and justify the minimal important change in individuals	n/a
	12.3		If the outcome data collected are continuous but will be analyzed as categorical (method of aggregation), specify the cutoff values to be used	n/a
	12.4		If outcome assessments will be performed at several time points after randomization, state the time points that will be used for analysis	29 - 34
	12.5		If a composite outcome is used, define all individual components of the composite outcome	n/a
Participant timeline	13	Time schedule of enrolment, interventions (including any run-ins and washouts), assessments, and visits for participants. A schematic diagram is highly recommended (see Figure)	-	32, 33
Sample size	14	Estimated number of participants needed to achieve study objectives and how it was determined, including clinical and statistical assumptions supporting any sample size calculations	-	43
	14.1		Define and justify the target difference between treatment groups (eg, the minimal important difference)	n/a
Recruitment	15	Strategies for achieving adequate participant enrolment to reach target sample size	-	12, 25 - 26
**Methods: Assignment of interventions (for controlled trials)**				
Allocation:				
Sequence generation	16a	Method of generating the allocation sequence (eg, computer-generated random numbers), and list of any factors for stratification. To reduce predictability of a random sequence, details of any planned restriction (eg, blocking) should be provided in a separate document that is unavailable to those who enrol participants or assign interventions	-	27

For peer review only - http://bmjopen.bmj.com/site/about/guidelines.xhtml

Section	Item No.	SPIRIT 2013 Item	SPIRIT-Outcomes 2022 item	Location Reported[b]
Allocation concealment mechanism	16b	Mechanism of implementing the allocation sequence (eg, central telephone; sequentially numbered, opaque, sealed envelopes), describing any steps to conceal the sequence until interventions are assigned	-	27 - 28
Implementation	16c	Who will generate the allocation sequence, who will enrol participants, and who will assign participants to interventions	-	27
Blinding (masking)	17a	Who will be blinded after assignment to interventions (eg, trial participants, care providers, outcome assessors, data analysts), and how	-	n/a
	17b	If blinded, circumstances under which unblinding is permissible, and procedure for revealing a participant's allocated intervention during the trial	-	n/a
**Methods: Data collection, management, and analysis**				
Data collection methods	18a	Plans for assessment and collection of outcome, baseline, and other trial data, including any related processes to promote data quality (eg, duplicate measurements, training of assessors) and a description of study instruments (eg, questionnaires, laboratory tests) along with their reliability and validity, if known. Reference to where data collection forms can be found, if not in the protocol	-	12, 26, 28, 30 - 34, 47
	18a.1		Describe what is known about the responsiveness of the study instruments in a population similar to the study sample	30
	18a.2		Describe who will assess the outcome (eg, nurse, parent)	33
	18b	Plans to promote participant retention and complete follow-up, including list of any outcome data to be collected for participants who discontinue or deviate from intervention protocols	-	22, 44

For peer review only - http://bmjopen.bmj.com/site/about/guidelines.xhtml

Section	Item No.	SPIRIT 2013 Item	SPIRIT-Outcomes 2022 item	Location Reported[b]
Data management	19	Plans for data entry, coding, security, and storage, including any related processes to promote data quality (eg, double data entry; range checks for data values). Reference to where details of data management procedures can be found, if not in the protocol	-	37, 39
Statistical methods	20a	Statistical methods for analysing primary and secondary outcomes. Reference to where other details of the statistical analysis plan can be found, if not in the protocol	-	45
	20a.1		Describe any planned methods to account for multiplicity in the analysis or interpretation of the primary and secondary outcomes (eg, coprimary outcomes, same outcome assessed at multiple time points, or subgroup analyses of an outcome)	n/a
	20b	Methods for any additional analyses (eg, subgroup and adjusted analyses)	-	n/a
	20c	Definition of analysis population relating to protocol non-adherence (eg, as randomised analysis), and any statistical methods to handle missing data (eg, multiple imputation)	-	45
**Methods: Monitoring**				
Data monitoring	21a	Composition of data monitoring committee (DMC); summary of its role and reporting structure; statement of whether it is independent from the sponsor and competing interests; and reference to where further details about its charter can be found, if not in the protocol. Alternatively, an explanation of why a DMC is not needed	-	9, 46
	21b	Description of any interim analyses and stopping guidelines, including who will have access to these interim results and make the final decision to terminate the trial	-	n/a
Harms	22	Plans for collecting, assessing, reporting, and managing solicited and spontaneously reported adverse events and other unintended effects of trial interventions or trial conduct	-	44 - 45

For peer review only

SPIRIT | CONSORT OUTCOMES
Reporting Guidelines for Trial Outcomes

Section	Item No.	SPIRIT 2013 Item	SPIRIT-Outcomes 2022 item	Location Reported[b]
Auditing	23	Frequency and procedures for auditing trial conduct, if any, and whether the process will be independent from investigators and the sponsor	-	42
**Ethics and dissemination**				
Research ethics approval	24	Plans for seeking research ethics committee/institutional review board (REC/IRB) approval	-	47
Protocol amendments	25	Plans for communicating important protocol modifications (eg, changes to eligibility criteria, outcomes, analyses) to relevant parties (eg, investigators, REC/IRBs, trial participants, trial registries, journals, regulators)	-	2
Consent or assent	26a	Who will obtain informed consent or assent from potential trial participants or authorised surrogates, and how (see Item 32)	-	24 - 25
	26b	Additional consent provisions for collection and use of participant data and biological specimens in ancillary studies, if applicable	-	n/a
Confidentiality	27	How personal information about potential and enrolled participants will be collected, shared, and maintained in order to protect confidentiality before, during, and after the trial	-	40, 47 - 48
Declaration of interests	28	Financial and other competing interests for principal investigators for the overall trial and each study site	-	n/a
Access to data	29	Statement of who will have access to the final trial dataset, and disclosure of contractual agreements that limit such access for investigators	-	48 - 49
Ancillary and post-trial care	30	Provisions, if any, for ancillary and post-trial care, and for compensation to those who suffer harm from trial participation	-	n/a
Dissemination policy	31a	Plans for investigators and sponsor to communicate trial results to participants, healthcare professionals, the public, and other relevant groups (eg, via publication, reporting in results databases, or other data sharing arrangements), including any publication restrictions	-	50
	31b	Authorship eligibility guidelines and any intended use of professional writers	-	50

For peer review only - http://bmjopen.bmj.com/site/about/guidelines.xhtml

Section	Item No.	SPIRIT 2013 Item	SPIRIT-Outcomes 2022 item	Location Reported[b]
	31c	Plans, if any, for granting public access to the full protocol, participant-level dataset, and statistical code	-	-
**Appendices**				
Informed consent materials	32	Model consent form and other related documentation given to participants and authorised surrogates	-	-
Biological specimens	33	Plans for collection, laboratory evaluation, and storage of biological specimens for genetic or molecular analysis in the current trial and for future use in ancillary studies, if applicable	-	n/a

[a]It is strongly recommended that this checklist be read in conjunction with the SPIRIT (Standard Protocol Items: Recommendations for Interventional Trials) Statement paper for important clarification on the items. Amendments to the protocol should be tracked and dated. The SPIRIT checklist is copyrighted by the SPIRIT Group under the Creative Commons "Attribution-NonCommercial-NoDerivs 3.0 Unported" license and is reproduced with permission.
[b]Indicates page numbers and/or manuscript location: to be completed by authors.

For peer review only
