# Supplementary material for: Pilot study to evaluate the use of remote patient monitoring to guide the timing of valve intervention in patients with severe asymptomatic aortic stenosis (APRAISE-AS): study protocol for a randomised controlled trial delivered in two tertiary cardiac centres in the UK
Source: BMJ Open. 2024 Jun 10;14(6):e086587. doi: 10.1136/bmjopen-2024-086587 (PMC11168151; doi:10.1136/bmjopen-2024-086587)
Supplement: Reviewer comments [file bmjopen-2024-086587.reviewer_comments.pdf]

PEER REVIEW HISTORY

BMJ Open publishes all reviews undertaken for accepted manuscripts. Reviewers are asked to complete a checklist review form (**http://bmjopen.bmj.com/site/about/resources/checklist.pdf**) and are provided with free text boxes to elaborate on their assessment. These free text comments are reproduced below.

ARTICLE DETAILS

**TITLE (PROVISIONAL)**	A pilot study to evaluate the use of remote patient monitoring to guide the timing of valve intervention in patients with severe asymptomatic aortic stenosis (APRAISE-AS): study protocol for a randomised controlled trial delivered in two tertiary cardiac centres in the United Kingdom
**AUTHORS**	Khan, Nazish; Steeds, Richard; Kyte, Derek; Fabritz, Larissa; Collis, Philip; Chua, Winnie; Stubbs, Clive; Mehta, Samir; Sun, Yongzhong; Nulty, Mary; Kirkham, Katie; Cotton, James

This article was not externally reviewed at BMJ Open. Protocols that have been independently assessed prior to submission to BMJ Open are usually fast-tracked to publication on the grounds that further substantial changes will not be possible. This independent assessment will usually be external, independent review for both a substantial grant award from a non-commercial or government funder and ethics approval.
